# Distinct Amino Acid Availability-Dependent Regulatory Mechanisms of MepS and MepM Levels in *Escherichia coli*

**DOI:** 10.3389/fmicb.2021.677739

**Published:** 2021-06-30

**Authors:** Yung Jae Kim, Byoung Jun Choi, Si Hyoung Park, Han Byeol Lee, Ji Eun Son, Umji Choi, Won-Jae Chi, Chang-Ro Lee

**Affiliations:** ^1^Department of Biological Sciences, Myongji University, Yongin, South Korea; ^2^Biological and Genetic Resource Assessment Division, National Institute of Biological Resource, Incheon, South Korea

**Keywords:** peptidoglycan hydrolase, peptidoglycan endopeptidase, MepS, MepM, Prc, NlpI, amino acid availability

## Abstract

Peptidoglycan (PG) hydrolases play important roles in various aspects of bacterial physiology, including cytokinesis, PG synthesis, quality control of PG, PG recycling, and antibiotic resistance. However, the regulatory mechanisms of their expression are poorly understood. In this study, we have uncovered novel regulatory mechanisms of the protein levels of the synthetically lethal PG endopeptidases MepS and MepM, which are involved in PG synthesis. A mutant defective for both MepS and MepM was lethal in an amino acid-rich medium, whereas it exhibited almost normal growth in a minimal medium, suggesting the expendability of MepS and MepM in a minimal medium. Protein levels of MepS and MepM dramatically decreased in the minimal medium. Although MepM was revealed as a substrate of Prc, a periplasmic protease involved in the proteolysis of MepS, only the decrease in the MepS level in the minimal medium was affected by the *prc* depletion. Phenotypic and biochemical analyses showed that the presence of aromatic amino acids in the medium induced the accumulation of MepS, but not MepM, while the presence of glutamate increased the level of MepM, but not MepS. Together, these results demonstrate that the protein levels of the two major PG endopeptidases are regulated in an amino acid availability-dependent manner, but their molecular mechanisms and signaling are significantly distinct.

## Introduction

Bacteria adapt to various environmental stresses through changes in gene expression, which induces optimal growth of bacteria and increases the survival rate. Among the regulatory mechanisms of gene expression, post-translational regulation exerted by proteases plays a crucial role in the regulation of cellular activities in bacteria. Although the functions of cytoplasmic proteases have been extensively studied, studies on the physiological roles of periplasmic proteases in Gram-negative bacteria are relatively rare.

Peptidoglycan (PG) hydrolases are periplasmic enzymes that catalyze the cleavage of various covalent bonds of PG, including β-1,4 glycosidic bonds, amide bonds between peptide side chains and N-acetylmuramic acid, and cross-links between peptide side chains. The cleavage of PG by PG hydrolases is necessary for diverse physiological processes, such as cytokinesis (Uehara et al., 2010; Weaver et al., 2019), PG synthesis (Singh et al., 2012; Lai et al., 2017; Chodisetti and Reddy, 2019; Park et al., 2020), quality control of PG (Cho et al., 2014), PG recycling (Mayer et al., 2019; Hernandez et al., 2020), the establishment of flagella and type VI secretion architecture (Nambu et al., 1999; Santin and Cascales, 2017), stress adaptation (Moll et al., 2015; Mueller and Levin, 2020), and antibiotic resistance (Dorr et al., 2015; Hugonnet et al., 2016).

Among PG hydrolases, PG endopeptidases catalyze the hydrolysis of cross-links between peptide side chains, which creates space for the insertion of a new PG strand and consequently stimulates PG synthesis (Burman and Park, 1984; Singh et al., 2012; Lai et al., 2017). In *Escherichia coli*, there are seven PG endopeptidases (Vermassen et al., 2019; Park et al., 2020). Our recent report based on phenotypic analysis showed that PG endopeptidases have distinct roles in penicillin-binding protein 1a (PBP1a)- and PBP1b-related functions, which are involved in PG synthesis (Park et al., 2020). Distinct roles of PG endopeptidases were also reported in *Lactobacillus plantarum* (Duchene et al., 2019). Deletion of PG endopeptidase MepM caused salt sensitivity, which was also observed in the mutant defective for PBP1a or PBP1b (Park et al., 2020). Deletion of PG endopeptidase MepS resulted in EDTA sensitivity and the growth defect on the NA medium at high temperature (Singh et al., 2012; Park et al., 2020). Depletion of MepM and MepS was synthetically lethal in the LB medium, and depletion of MepM, MepS, and MepH was synthetically lethal in the minimal medium (Singh et al., 2012).

Despite the physiological importance of PG endopeptidases, studies on the regulatory mechanisms of PG hydrolase expression are rare. In *E. coli*, MepS in the outer membrane is subject to degradation by a periplasmic protease, Prc, and the degradation is enhanced by an outer membrane adaptor protein, NlpI (Singh et al., 2015). A similar mechanism has been reported in *Pseudomonas aeruginosa* (Srivastava et al., 2018). In *Vibrio cholerae*, the expression of the metal chelator-resistant PG endopeptidase, ShyB, is induced under zinc starvation in a Zur-dependent manner (Murphy et al., 2019).

In this study, we investigated the regulatory mechanisms of the expression of two major PG endopeptidases, MepS and MepM, in *E. coli*. A Δ*mepS* Δ*mepM* mutant was lethal in the amino acid-rich medium, but not in the minimal medium, and the amount of MepS and MepM dramatically increased in an amino acid-rich medium. *In vitro* and *in vivo* experiments showed that MepM, similar to MepS, was also degraded by Prc. However, only the accumulation of MepS in the amino acid-rich medium was subjected to Prc-dependent regulation. Furthermore, we showed that the presence of aromatic amino acids in the medium induced the accumulation of MepS, but not MepM, and the presence of glutamate increased the MepM level, but not MepS. In summary, we demonstrated that MepS and MepM are required for normal growth under the amino acid-rich conditions, and their protein levels are enhanced under these conditions through distinct signaling and mechanisms.

## Materials and Methods

### Bacterial Strains, Plasmids, and Culture Conditions

All strains and primers used in this study are listed in Supplementary Tables 1, 2, respectively. *E. coli* MG1655 strains were cultured at 37°C in the Luria–Bertani (LB) medium or the M9 minimal medium containing 0.2% glucose. In an M9 minimal medium containing glucose, 0.2% casamino acids or amino acids (10 or 20 mM each) were added when necessary. Antibiotics, such as chloramphenicol (5 μg/ml), kanamycin (50 μg/ml), ampicillin (100 μg/ml), and tetracycline (10 μg/ml), were added to the culture medium when necessary.

All deletion mutants were constructed using λ red recombinase, as described previously, with some modifications (Datsenko and Wanner, 2000). To construct the strain chromosomally expressing MepS with a 3 × Flag epitope at its C-terminus, the region covering the *3* × *Flag* gene and the chloramphenicol resistance gene were amplified using the plasmid pBAD-Flag with the *3* × *Flag* gene as a template (Park et al., 2020). After PCR purification, the template plasmids were digested with DpnI overnight. DpnI-treated PCR products were electroporated into MG1655 cells harboring the plasmid pKD46 and integrated into the 3' end of the *mepS* gene. Recombinants were selected on LB plates containing chloramphenicol. Insertion of the *3* × *Flag* gene and the chloramphenicol resistance gene was confirmed using PCR with the primer sets presented in Supplementary Table 2. Other strains that chromosomally express proteins with the 3 × Flag epitope at their C-terminus were constructed using the same method.

To construct the plasmid pET-His-Prc(Δss), the *prc* gene without the region encoding the signal sequence (amino acids 1-22) was amplified via PCR using the genomic DNA of MG1655 cells as a template and a primer set having sequences for recombination with the plasmid vector at the 5' end (see Supplementary Table 2). After PCR purification, PCR products were cloned into the plasmid pET28a digested by NdeI and BamHI restriction enzymes via the recombination between overlapping sequences using In-Fusion^®^ cloning (Clontech, USA). Plasmid construction was confirmed by sequencing analysis. Using the same method, the plasmids for the expression of other proteins, including NlpI without the signal sequence (amino acids 1–20), MepS without the signal sequence (amino acids 1–27), MepM without the transmembrane domain (amino acids 1–40), MepH without the signal sequence (amino acids 1–27), and PbpG without the signal sequence (amino acids 1–29), were constructed.

### Purification of Proteins and *in vitro* Proteolysis Assay

His-tagged proteins were overexpressed in ER2566 cells harboring a plasmid for target protein expression. Cells cultured in the LB medium overnight were inoculated in 200 ml of the LB medium and cultured at 37°C until the *OD*_600nm_ was 0.6. After the addition of 1 mM isopropyl-β-d-1-thiogalactopyranoside (IPTG), cells were cultured at 16°C overnight. Harvested cells were resuspended in 3 ml of buffer A (50 mM Tris-HCl [pH 8.0] and 200 mM NaCl) and disrupted by a French pressure cell at 10,000 psi. The cell lysate was centrifuged at 8,000 × *g* for 20 min at 4°C, and only the supernatant was transferred to 500 μl of a TALON metal affinity resin (Clontech, USA) equilibrated with buffer A. After mixing at 4°C for 10 min, the flow-through was removed. After washing three times with 4 ml of buffer A, proteins bound to the resins were eluted with buffer A containing 200 mM imidazole, and eluted solutions were dialyzed overnight with 2 L of 50 mM Tris-HCl (pH 8.0) and 50 mM NaCl.

For the *in vitro* proteolysis assay, purified Prc (0.64 μg) and NlpI (1 or 0.3 μg) were mixed with PG endopeptidases (1.2 or 0.3 μg). The reaction buffer consisted of 50 mM Tris-HCl (pH 8.0) and 100 mM NaCl. The samples were incubated at 37°C for the indicated times. After adding 1 × SDS protein sample buffer, proteins were separated using 4–20% gradient acrylamide gel. Proteins were detected by staining with Coomassie Brilliant Blue R.

### Detection of Intracellular Levels of PG Endopeptidases

Intracellular protein levels were measured using the strain with the integration of the *3* × *Flag* gene at the 3' end of each gene. Cells grown to the early exponential phase (*OD*_600*nm*_ = 0.4) in the LB or M9 minimal medium were harvested by centrifugation at 14,000 rpm. After adding 1 × SDS protein sample buffer, the samples were boiled at 100°C for 10 min. After vortexing and cooling, 5 × 10^7^ or 5 × 10^8^ cells were loaded onto 4–20% gradient acrylamide gel and run at 150 V for 90 min. Proteins in the acrylamide gel were transferred to a nitrocellulose membrane, which was then blocked with PBS-T buffer containing 1% polyvinylpyrrolidone. Immunoblotting was performed according to standard procedures using anti-Flag (Santa Cruz Biotechnology, USA), anti-DnaK (Abcam, UK), and anti-FtsZ (Agrisera, Sweden) antibodies. DnaK and FtsZ were used as the loading controls.

### Quantitative Real-Time PCR

Transcript levels of the *mepM* and *mepS* genes were measured using quantitative real-time PCR. MG1655 cells were grown at 37°C in the LB or M9 minimal medium to mid-exponential phase (*OD*_600*nm*_ = 0.8), and 10^9^ cells were harvested. After treatment with lysozyme, mRNA was extracted using a Qiagen RNA extraction kit (Qiagen, USA) according to the standard protocol. cDNA was synthesized from DNase I-treated total RNA using the cDNA EcoDry Premix (Clontech, USA). Real-time PCR was performed using the CFX96 Real-Time System (Bio-Rad, USA) and primer sets (see Supplementary Table 2) designed for amplification of the 5' regions of *mepM* and *mepS*. The relative expression level was calculated as the difference between the threshold cycles of the target genes and the threshold cycle of the reference gene (16S rRNA) for each sample.

### Binding Test Between NlpI and PG Endopeptidases

Interactions between NlpI and PG endopeptidases were examined using pull-down experiments. His-tagged NlpI(Δss) was purified using the standard method described above. Non-His-tagged PG endopeptidases were overexpressed in ER2566 cells harboring a plasmid for expression of the target protein. Cells cultured in the LB medium overnight were inoculated in 200 ml of the LB medium and cultured at 37°C until the *OD*_600nm_ was 0.6. After the addition of 1 mM IPTG, the cells were cultured overnight at 16°C. Harvested cells were resuspended in 3 ml of buffer A and disrupted by a French pressure cell at 10,000 psi. Cell lysates were centrifuged at 8,000 × *g* for 20 min at 4°C, and only the supernatant was transferred to 150 μl of a TALON metal affinity resin equilibrated with buffer A. One resin contained only the supernatant of PG endopeptidases, whereas the other contained purified His-NlpI(Δss) and the supernatant of PG endopeptidases. After mixing at 4°C for 25 min, the flow-through was removed. After washing three times with 1 ml of buffer A, 100 μl of 2 × SDS protein sample buffer was added, and the samples were boiled at 100°C for 5 min. After vortexing and cooling, 20 μl of each sample was loaded onto 4–20% gradient acrylamide gel, followed by electrophoresis. Proteins were detected by staining with Coomassie Brilliant Blue R.

## Results

### MepS and MepM Are Not Necessary for *E. coli* Growth in M9 Minimal Medium

**Figure 1:**
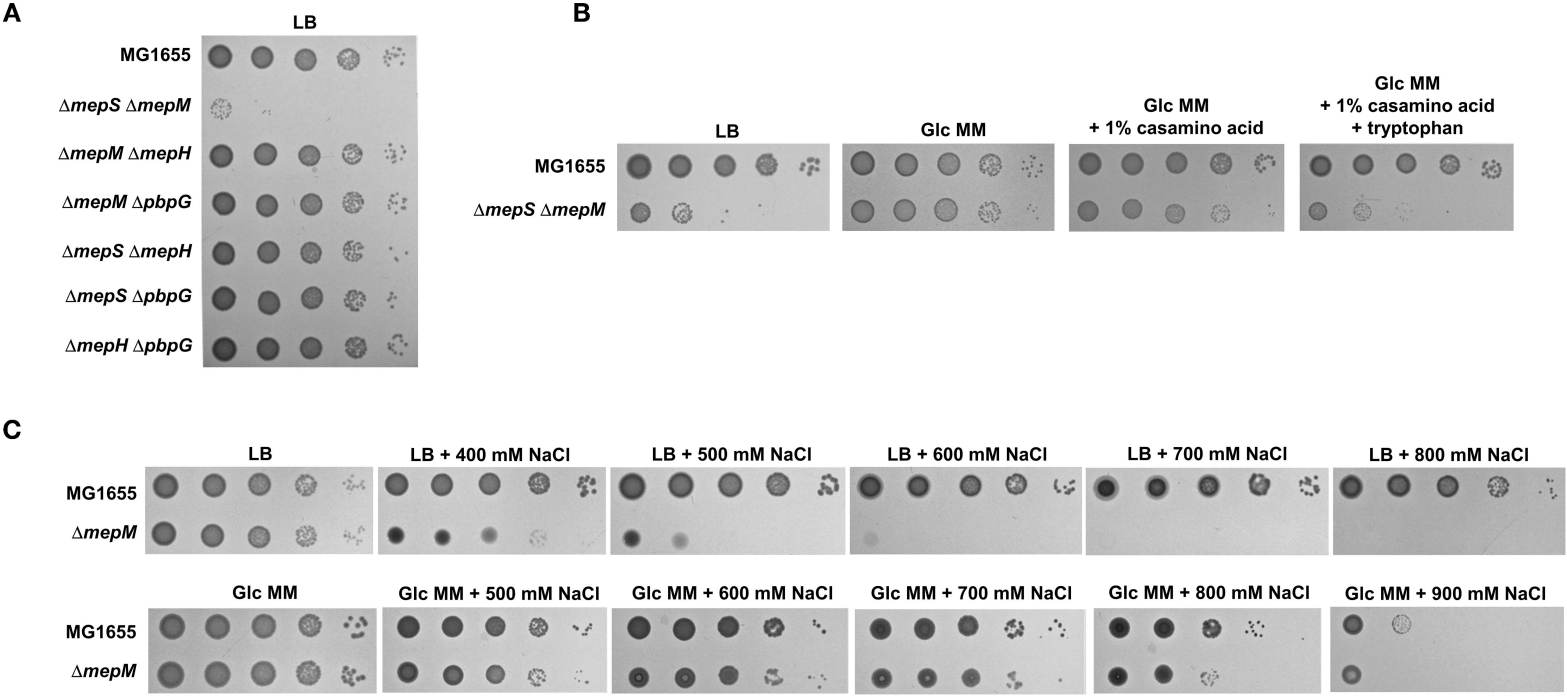
MepS and MepM are required for bacterial growth only in the LB medium. **(A)** Only the *mepS mepM* double mutant is lethal in the LB medium. Indicated strains were serially diluted from 10^8^ to 10^4^ cells/ml in 10-fold steps and spotted onto an LB plate. **(B)** Normal growth of the *mepS mepM* double mutant in the M9 minimal medium. Indicated strains were serially diluted from 10^8^ to 10^4^ cells/ml in 10-fold steps and spotted onto an LB plate (LB), an M9 minimal medium plate containing 0.2% glucose (Glc MM), an M9 minimal medium plate containing 0.2% glucose and 1% casamino acid, or an M9 minimal medium plate containing 0.2% glucose, 1% casamino acid, and 10 mM tryptophan. **(C)** Salt sensitivity of the *mepM* mutant is abolished in the M9 minimal medium. Indicated strains were serially diluted from 10^8^ to 10^4^ cells/ml in 10-fold steps and spotted onto LB plates containing the indicated concentrations of NaCl or M9 minimal medium plates containing 0.2% glucose and the indicated concentrations of NaCl.

Previous studies have demonstrated that MepS and MepM are two major PG endopeptidases, and the *mepS mepM* double mutant is lethal in the LB medium (Singh et al., 2012; Park et al., 2020). To investigate this issue in more detail, we constructed various double mutants for four PG endopeptidases, namely, MepM, MepS, MepH, and PbpG. Apart from the *mepS mepM* double mutant, the growth of all double mutants was comparable to that of the wild-type strain (Figure 1A). The growth defect of the *mepS mepM* double mutant was complemented by the ectopic expression of MepS or MepM (Supplementary Figure 1). These results support that MepS and MepM are two major PG endopeptidases that are required for *E. coli* growth in the LB medium. Interestingly, the requirement of MepS and MepM was almost abolished in the M9 minimal medium containing glucose; the growth of the *mepS mepM* double mutant and wild-type strains was almost similar in the M9 minimal medium (Figure 1B; Supplementary Figure 2). This result is consistent with data from a previous report (Singh et al., 2012). In a previous study, we found a salt-sensitive phenotype in the *mepM* mutant (Park et al., 2020). A similar reversion of the phenotype was detected in the salt-sensitive phenotype of the *mepM* mutant (Figure 1C); the salt-sensitive phenotype of the *mepM* mutant was not observed in the M9 minimal medium. Taken together, these results strongly imply that MepS and MepM are required for bacterial growth only in the LB medium, but not essential in the M9 minimal medium containing glucose.

### Protein Levels of MepS and MepM Are Strongly Diminished in Minimal Medium

**Figure 2:**
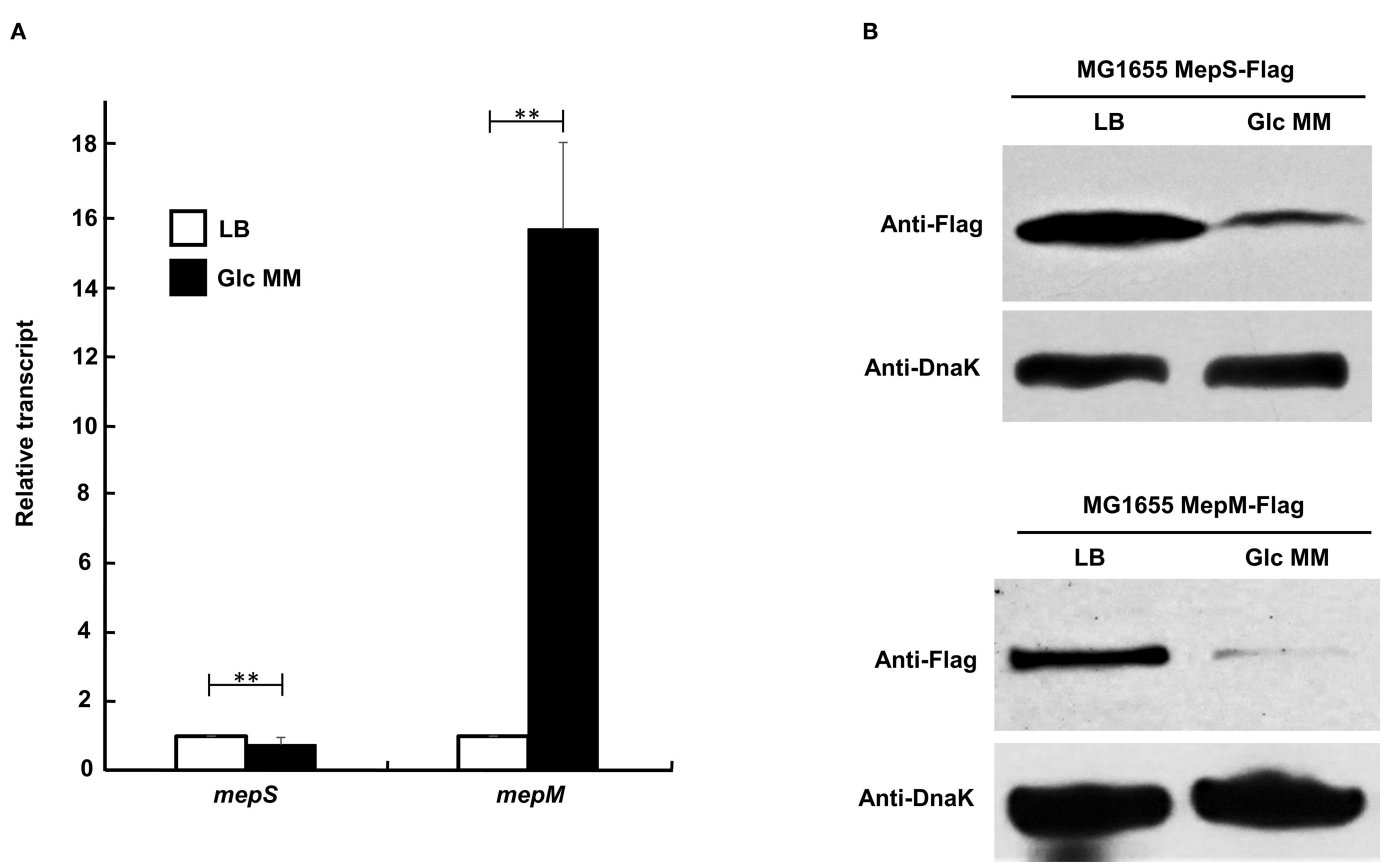
Changes in mRNA and protein levels of MepS and MepM in LB and M9 minimal media. **(A)** Transcript levels of the *mepS* and *mepM* genes in LB (white bars) and M9 minimal media (black bars). mRNA was extracted from MG1655 cells grown in the LB or M9 minimal medium containing 0.2% glucose to early exponential phase (OD_600nm_ = 0.4). Data were obtained from three independent experiments. mRNA levels were normalized to the concentration of 16S rRNA. **p < 0.01 **(B)** Protein levels of MepS and MepM in the LB or M9 minimal medium. Western blot analysis with anti-Flag and anti-DnaK antibodies was performed using 5 x 10^7^ cells in MG1655 MepS-Flag or 5 x 10^8^ cells in MG1655 MepM-Flag, grown in the LB or M9 minimal medium containing 0.2% glucose (Glc MM) to the early exponential phase (*OD*_600*nm*_ = 0.4). DnaK was used as the loading control.

Because phenotypic analysis strongly suggests the unnecessity of MepS and MepM for bacterial growth in the minimal medium, we wondered whether the expression of MepS and MepM is diminished in the minimal medium compared to that in the LB medium. Unexpectedly, the transcriptional level of the *mepS* gene slightly decreased in the minimal medium compared to that in the LB medium, whereas the transcriptional level of the *mepM* gene increased by more than 15-fold in the minimal medium (Figure 2A). MepS is subjected to strong post-translational regulation by an ATP-independent periplasmic tail-specific protease, Prc (Singh et al., 2015; Su et al., 2017). Therefore, we analyzed the protein levels of MepS and MepM in the minimal medium. Notably, a strong decrease in the protein level in the minimal medium compared to that in the LB medium was found in MepM as well as in MepS (Figure 2B). These results imply that MepS and MepM are regulated by strong regulatory mechanisms. The strong decrease in MepS and MepM levels in the minimal medium prompts us to investigate the effect of MepS or MepM overexpression in the minimal medium on the bacterial growth. The overexpression of MepS or MepM using the pBAD plasmid with an arabinose-inducible promoter induced the significant growth defect in the minimal medium, but this significant growth defect was not observed in the LB medium (Supplementary Figure 3). These results imply that the tight regulation of MepS and MepM levels along culture media is necessary for the normal bacterial growth.

### MepM Is an NlpI-Independent Substrate of Prc

**Figure 3:**
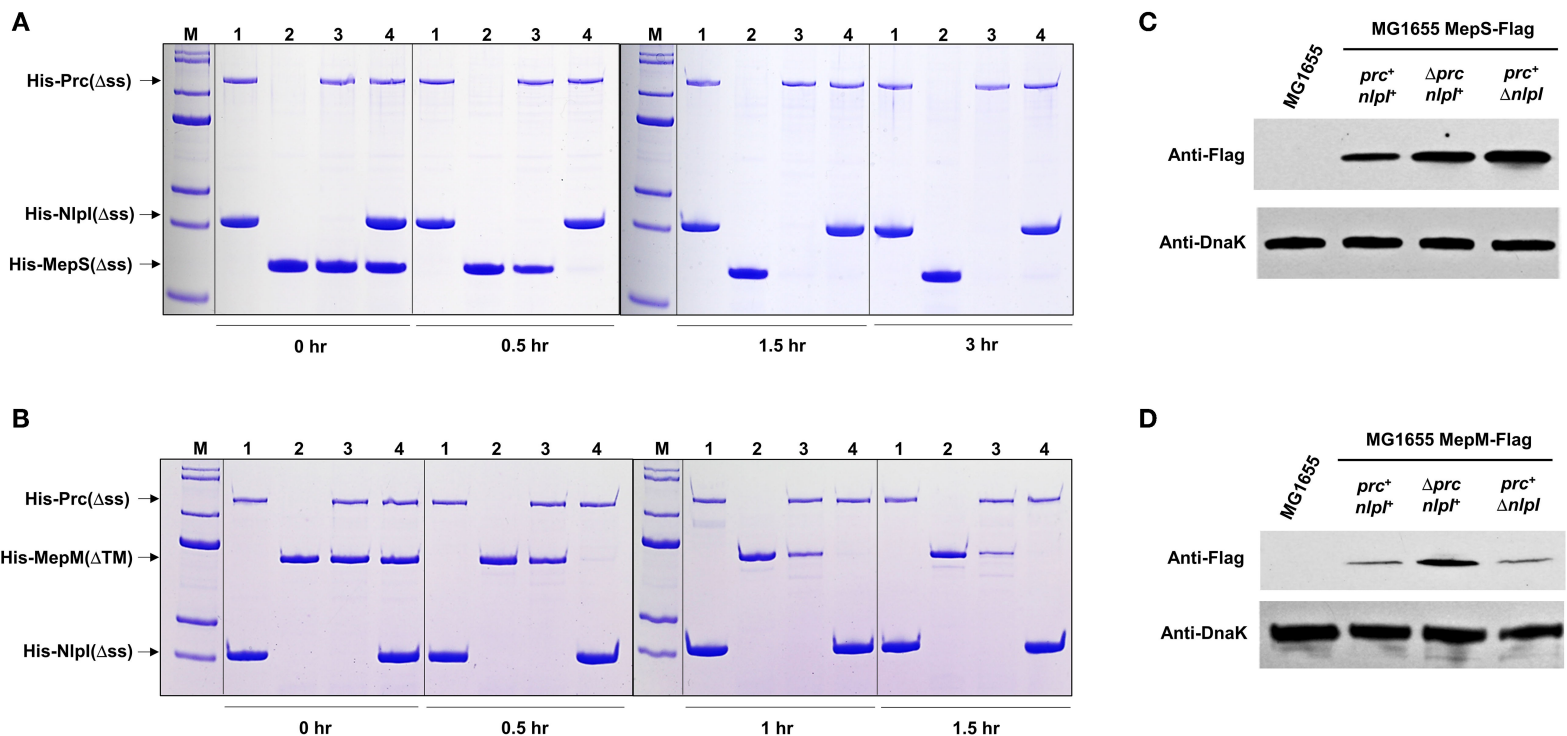
MepM is another substrate of Prc. **(A,B)** *In vitro* proteolysis assay of His-MepS and His-MepM by Prc. Purified MepS **(A)** or MepM **(B)** was incubated with Prc in the presence or absence of NlpI at 37°C for the indicated times. Samples were analyzed by SDS-PAGE and stained with Coomassie Brilliant Blue R. Lane M, EzWayTM Protein Blue MW Marker (KOMA Biotech., Korea); lane 1, His-Prc(Δss) and His-NlpI(Δss); lane 2, His-MepS(Δss) or His-MepM(ΔTM); lane 3, His-Prc(Δss) and His-MepS(Δss) or His-MepM(ΔTM); lane 4, His-Prc(Δss), His-NlpI(Δss), and His-MepS(Δss) or His-MepM(ΔTM). **(C,D)** Intracellular levels of MepS and MepM. Indicated strains were grown in the LB medium to the exponential phase (*OD*_600*nm*_ = 0.8). Harvested cells (5 x 10^7^ cells in MG1655 MepS-Flag or 5 x 10^8^ cells in MG1655 MepM-Flag) were used to determine the intracellular levels of MepS **(C)** and MepM **(D)** using an anti-Flag antibody. DnaK was used as the loading control.

In a previous study, we found an EDTA-sensitive phenotype in the *mepS* mutant (Park et al., 2020). This phenotype was restored by the overexpression of MepH, PbpG, and MepM, as well as MepS. Interestingly, the EDTA sensitivity of the *mepS* mutant was also partially suppressed by the deletion of Prc (Supplementary Figure 4), implying the Prc-mediated regulation of MepH, PbpG, or MepM. To assess this possibility, we purified various PG endopeptidases and performed an *in vitro* protein degradation assay. As expected, MepS was degraded by Prc, and the degradation rate was strongly enhanced in the presence of NlpI (Figure 3A), which is known as a partner lipoprotein of Prc (Singh et al., 2015). MepM and PbpG were also degraded by Prc, and their degradation rates were enhanced in the presence of NlpI (Figure 3B; Supplementary Figure 5). However, MepH was not degraded by Prc, even in the presence of NlpI (Supplementary Figure 5). Noteworthily, the degradation rate of PbpG by Prc was significantly slower than that of MepS and MepM, regardless of the presence of NlpI.

To determine whether the proteolysis of MepM and PbpG by Prc occurs in cells, western blot analysis of these proteins was performed. The protein levels of MepS and MepM increased in the *prc* mutant, whereas the protein level of PbpG was hardly affected by the deletion of the *prc* gene (Figures 3C,D; Supplementary Figure 5), indicating that PbpG is not a substrate of Prc in *E. coli*. Deletion of the *nlpI* gene increased the expression of MepS up to a level comparable to that of the *prc* mutant, but the protein level of MepM did not increase in the *nlpI* mutant (Figures 3C,D). To analyze the discrepancy between *in vivo* and *in vitro* experiments with MepM, we examined the interaction between NlpI and PG endopeptidases. NlpI is known to enhance MepS degradation by Prc through direct interaction with MepS (Su et al., 2017). In our pull-down experiments, a tight interaction between NlpI and MepS was detected (Supplementary Figure 6). However, NlpI did not interact with other PG endopeptidases, including MepM. These results imply that the effect of NlpI on Prc-mediated proteolysis of MepM in *in vitro* experiments might be indirect. Purified Prc could be self-degraded at 37°C, and the presence of NlpI weakened the self-degradation (Figure 3A; Supplementary Figure 5), which is consistent with the previous data (Singh et al., 2015). Therefore, the effect of NlpI can be caused by the decrease in degraded or inactive forms of Prc. Even though NlpI directly activates the proteolysis of MepM, this effect may not occur in cells, owing to the discrepancy in cellular localization of NlpI and MepM. NlpI is an outer membrane lipoprotein (Su et al., 2017), whereas MepM is an inner membrane protein (Park et al., 2020). In summary, these data demonstrate that MepM is another substrate of Prc, but MepM degradation by Prc is not activated by NlpI.

### Protein Levels of MepS and MepM Decrease in the Minimal Medium Through Prc-Dependent and -Independent Mechanisms, Respectively

**Figure 4:**
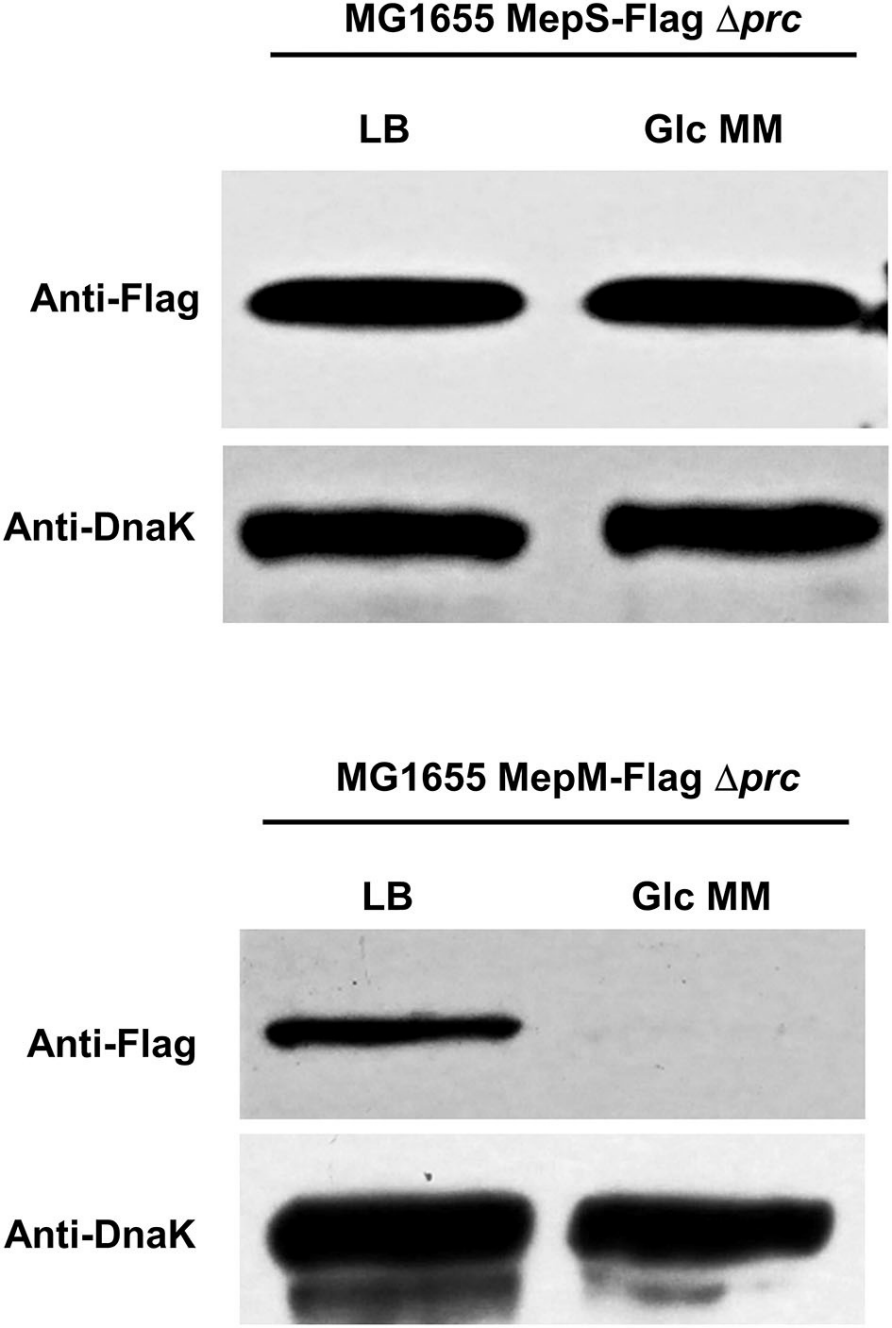
Effect of Prc on the protein levels of MepS and MepM in the M9 minimal medium. Indicated strains were grown in the LB or M9 minimal medium containing glucose (Glc MM) to the early exponential phase (*OD*_600*nm*_ = 0.4). Harvested cells (5 x 10^7^ cells in MG1655 MepS-Flag or 5 x 10^8^ cells in MG1655 MepM-Flag) were used to determine the intracellular levels of MepS and MepM using an anti-Flag antibody. DnaK was used as the loading control.

Given that both MepS and MepM are substrates of Prc, we wondered whether the decreased levels of the two proteins in the minimal medium are caused by Prc. Intriguingly, in the context of the *prc* deletion, the decrease in the protein level of MepS in the minimal medium was completely abolished, whereas the expression pattern of MepM was hardly affected by the *prc* deletion (Figure 4), indicating that the protein levels of MepS and MepM were diminished in the minimal medium by Prc-dependent and -independent mechanisms, respectively. The decrease in the MepS level in the minimal medium by Prc prompts us to investigate the effect of the *prc* deletion on the bacterial growth in the minimal medium. The *prc* mutant exhibited a slight growth defect in the minimal medium, but this growth defect was not observed in the LB medium (Supplementary Figure 7), implying that the regulation of MepS by Prc is required for the optimal growth in the minimal medium.

### MepS and MepM Are Essential for Bacterial Growth Under Amino Acid-Rich Condition

*E. coli* cells display a large difference in the growth rate between LB and M9 minimal media. To determine whether the growth rate affects the necessity of MepS and MepM, we examined the growth of the *mepS mepM* double mutant in the LB medium in relation with growth temperature. Although the growth rate of *E. coli* cells dramatically decreased as the temperature decreased, the growth defect of the *mepS mepM* double mutant was not recovered at all (Supplementary Figure 8A), indicating that the lethality of the *mepS mepM* double mutant in the LB medium was not directly due to the higher growth rate in the LB medium compared to that in the minimal medium.

The LB medium is different from the M9 minimal medium containing glucose in various aspects, such as carbon and nitrogen sources, the presence of amino acids and cofactors, salt concentrations, and metal concentrations. As glucose largely affects bacterial physiology, including gene expression (Deutscher et al., 2006; Park et al., 2013), we examined the effect of glucose addition on the LB medium. Glucose addition hardly affected the lethality of the *mepS mepM* double mutant in the LB medium (Supplementary Figure 8A). Salt concentration did not affect the lethality (Supplementary Figure 8A). Next, we examined the effects of the presence of amino acids. The *mepS mepM* double mutant showed almost normal growth in the minimal medium, but the addition of casamino acids in the minimal medium induced a slight growth defect in the mutant strain (Figure 1B). Tryptophan is known to lack in casamino acids due to its destruction by acid (Mueller and Johnson, 1941). Notably, when tryptophan and casamino acid were added to the minimal medium, the growth defect of the *mepS mepM* double mutant was almost similar to that in the LB medium (Figure 1B), indicating that the presence of amino acids, especially tryptophan, affects the necessity of MepS and MepM.

**Figure 5:**
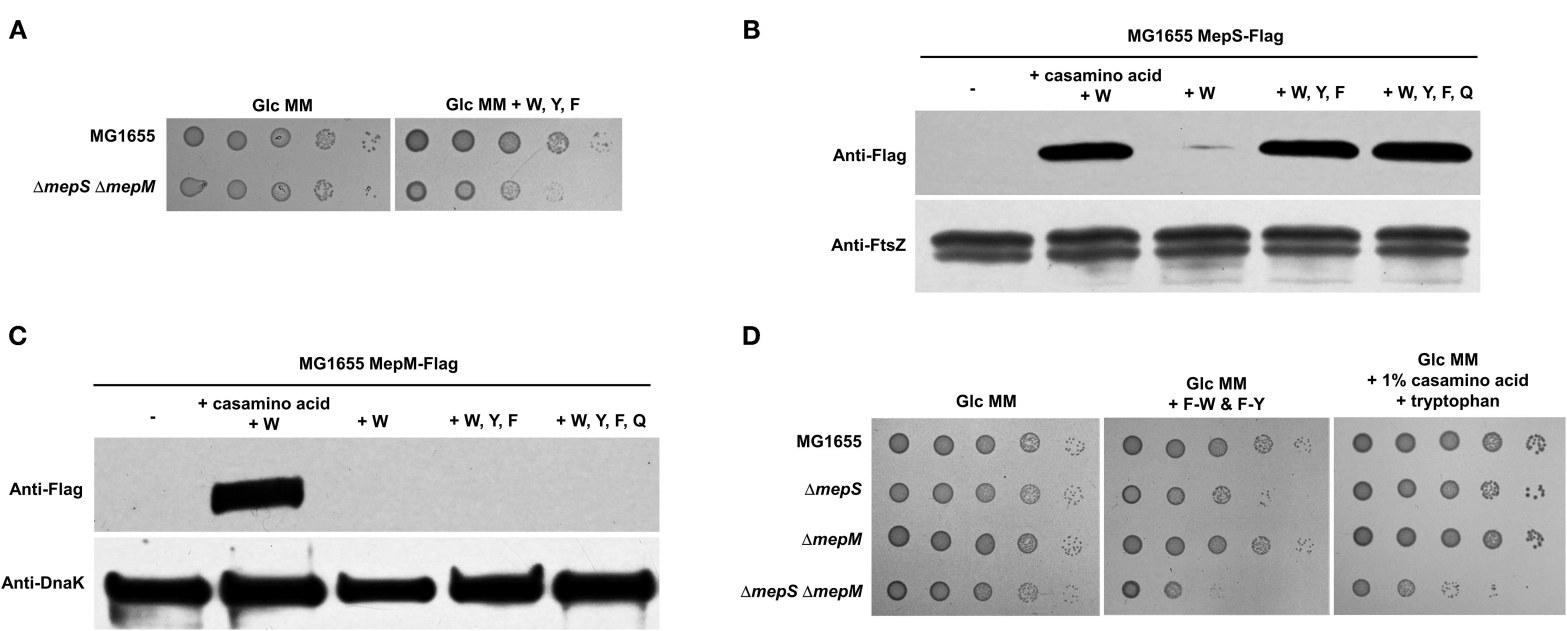
Effect of aromatic amino acids on the protein levels of MepS and MepM. **(A)** Effect of the addition of aromatic amino acids on the growth of the *mepS mepM* double mutant in the M9 minimal medium. Indicated strains were serially diluted from 10^8^ to 10^4^ cells/ml in 10-fold steps and spotted onto a glucose M9 minimal medium plate (Glc MM) and a glucose M9 minimal medium plate containing tryptophan (W), phenylalanine (F), and tyrosine (Y) (10 mM each). **(B)** Effect of aromatic amino acids on the protein level of MepS. MG1655 MepS-Flag cells were grown to the early exponential phase (*OD*_600*nm*_ = 0.4) in the glucose M9 minimal medium containing 1% casamino acids or/and the indicated amino acids (10 mM each). Harvested cells (5 x 10^7^ cells) were used to determine the intracellular levels of MepS using an anti-Flag antibody. DnaK was used as the loading control. W, tryptophan; Y, tyrosine; F, phenylalanine; Q, glutamine. **(C)** Effect of aromatic amino acids on the protein level of MepM. MG1655 MepM-Flag cells were grown to the early exponential phase (*OD*_600*nm*_ = 0.4) in the glucose M9 minimal medium containing 1% casamino acids or/and the indicated amino acids (10 mM each). Harvested cells (5 x 10^8^ cells) were used to determine the intracellular levels of MepM using an anti-Flag antibody. **(D)** The *mepS* mutant phenocopies the *mepS mepM* double mutant only in the minimal medium containing aromatic amino acids. Indicated strains were serially diluted from 10^8^ to 10^4^ cells/ml in 10-fold steps and spotted onto a glucose M9 minimal medium plate, a glucose M9 minimal medium plate containing phenylalanine-tryptophan dipeptide (F-W) and phenylalanine-tyrosine dipeptide (F-Y) (each 3 mM), and a glucose M9 minimal medium plate containing 1% casamino acids and tryptophan (10 mM).

Given that tryptophan strongly affected the growth of the *mepS mepM* double mutant in the presence of casamino acids (Figure 1B), a mixture of tryptophan and other amino acid(s) could induce growth defects in the *mepS mepM* double mutant. Because tryptophan is an aromatic amino acid, we examined whether the mixture of aromatic amino acids affected the growth of the *mepS mepM* double mutant. The addition of an aromatic amino acid mixture induced a significant growth defect (Figure 5A), albeit weaker than in the case of casamino acids containing tryptophan. Therefore, these results show that the presence of aromatic amino acids partly triggers growth defects in the *mepS mepM* double mutant. Interestingly, when histidine was added to the minimal medium, the *mepS mepM* double mutant grew faster than the wild-type cells (Supplementary Figure 8B). Although we do not know the reason for this phenomenon, this result at least supports the model that amino acids are associated with the lethality of the *mepS mepM* double mutant.

### Protein Levels of MepS and MepM Increase in the Presence of Amino Acids

We wondered whether the presence of amino acids affected the protein levels of MepS and MepM. As expected, the mixture of casamino acids and tryptophan strongly induced the accumulation of both MepS and MepM, whereas tryptophan alone did not increase the levels of these two proteins (Figures 5B,C). We also examined the effects of the aromatic amino acids. Notably, the mixture of aromatic amino acids strongly increased the level of MepS up to a level comparable to that of the mixture of tryptophan and casamino acids, whereas it hardly affected the level of MepM (Figures 5B,C). Although both MepS and MepM levels are increased by the mixture of amino acids, specific amino acids that trigger their expressions seem to be different.

In the presence of aromatic amino acids, the protein level of MepS increases and that of MepM is very low. Therefore, the *mepS* mutant could phenocopy the *mepS mepM* double mutant in the presence of aromatic amino acids. Because the *mepS mepM* double mutant showed a growth defect in the minimal medium containing aromatic amino acids (Figure 5A), we examined the growth of the *mepS* mutant in the minimal medium containing aromatic amino acids. Notably, the *mepS* mutant exhibited growth defects only in the minimal medium containing aromatic amino acids, whereas the *mepS mepM* double mutant showed growth defects both in the minimal medium containing aromatic amino acids and in the minimal medium containing all amino acids (Figure 5C). In other words, only in the presence of aromatic amino acids the *mepS* mutant phenocopied the *mepS mepM* double mutant. These phenotypes support the hypothesis that the presence of aromatic amino acids induces only the accumulation of MepS.

**Figure 6:**
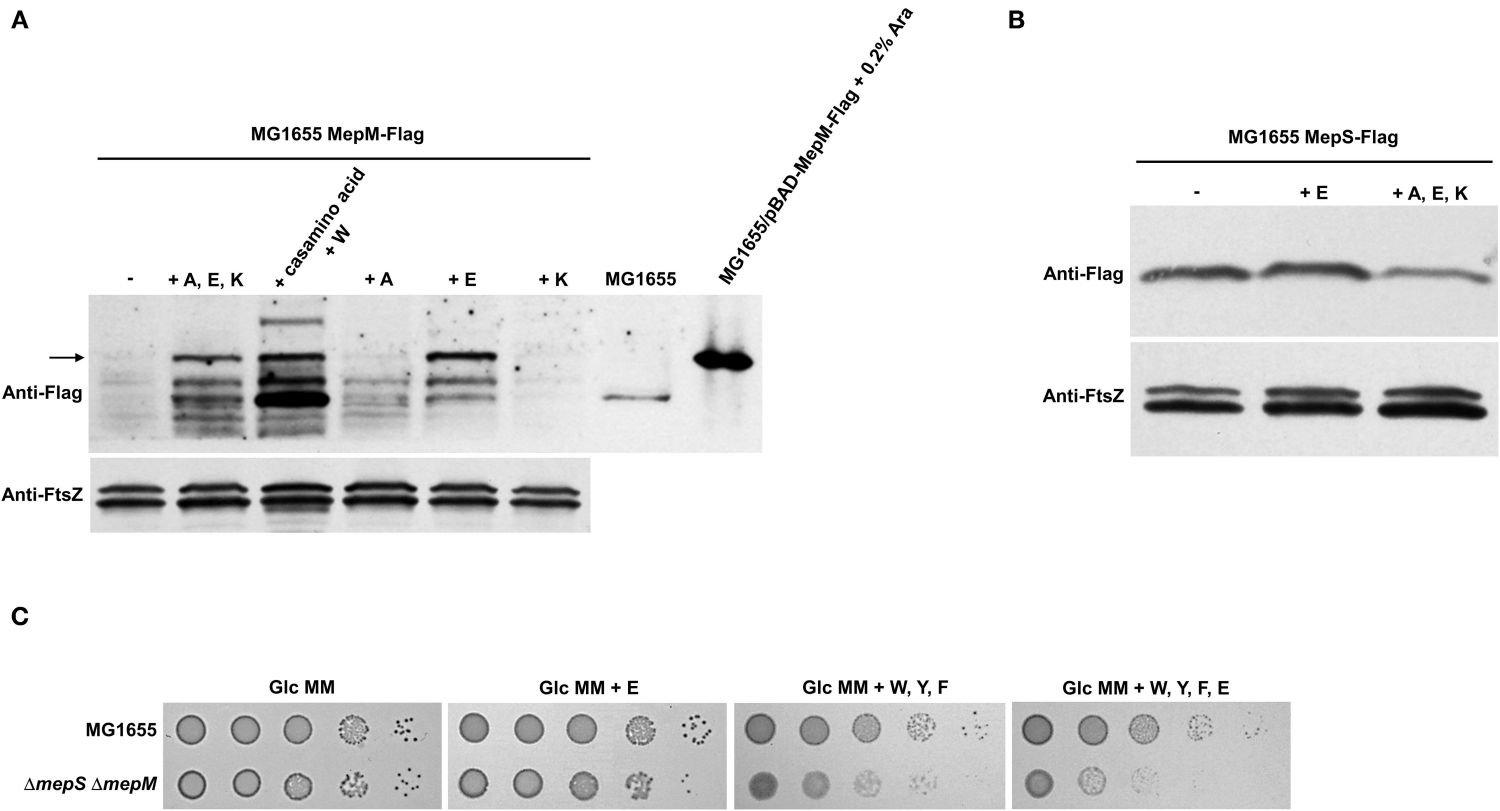
Effect of glutamate on the protein levels of MepS and MepM. **(A)** Effect of glutamate on the protein level of MepM. MG1655 MepS-Flag cells were grown to the early exponential phase (*OD*_600*nm*_ = 0.4) in the glucose M9 minimal medium containing 1% casamino acids or/and the indicated amino acids (10 mM each). Harvested cells (2 x 10^7^ cells in MG1655/pBAD-MepM-Flag and 2 x 10^8^ cells in other strains) were used to determine the intracellular levels of MepM using an anti-Flag antibody. FtsZ was used as the loading control. The arrow indicates MepM proteins. A, alanine; E, glutamate; K, lysine; W, tryptophan; Ara, arabinose. **(B)** Effect of amino acids on the protein level of MepS. MG1655 MepS-Flag cells were grown to the early exponential phase (*OD*_600*nm*_ = 0.4) in the glucose M9 minimal medium or glucose M9 minimal media containing indicated amino acids (10 mM each). Harvested cells (2 x 10^8^ cells) were used to determine the intracellular levels of MepS using an anti-Flag antibody. **(C)** Effect of the addition of amino acids on the growth of the *mepS mepM* double mutant in the M9 minimal medium. Indicated strains were serially diluted from 10^8^ to 10^4^ cells/ml in 10-fold steps and spotted onto a glucose M9 minimal medium plate (Glc MM) and glucose M9 minimal medium plates containing indicated amino acids (10 mM each). W, tryptophan; F, phenylalanine; Y, tyrosine; E, glutamate.

To identify amino acids that regulate the MepM level, we tested the effect of PG pentapeptide-related amino acids, including alanine, glutamate, and lysine, on the protein level of MepM. The presence of three amino acids increased the MepM level, and this effect was caused by the presence of glutamate (Figure 6A). Unlike MepM, the protein level of MepS was not affected by the presence of glutamate (Figure 6B). Given that the presence of aromatic amino acids induced the growth defect of the *mepS mepM* double mutant in the minimal medium, we wondered the effect of glutamate on the growth of the *mepS mepM* double mutant. The presence of glutamate alone very slightly inhibited the growth of the *mepS mepM* double mutant in the minimal medium, but glutamate evidently amplified the effect of aromatic amino acids (Figure 6C). Therefore, these results indicate that the presence of amino acids increases the protein levels of MepS and MepM, which is necessary for the normal growth of cells under amino acid-rich conditions; however, amino acids affecting the levels of the two proteins are distinct.

## Discussion

PG synthesis and degradation are essential but fluctuating processes that are necessary for maintaining the growth and survival of bacteria. Although PG hydrolases play a pivotal role in these processes, the regulation of their expression is poorly understood. In this study, we revealed amino acid availability-dependent regulation of PG endopeptidases. MepS and MepM are required for bacterial growth in an amino acid-rich environment; therefore, their protein levels increase in the amino acid-rich medium and decrease in the minimal medium. This switching of the protein level was mediated by Prc in MepS, whereas MepM was controlled by an unknown Prc-independent mechanism. Among the amino acids, the presence of aromatic amino acids increases the level of MepS, but not MepM, whereas the presence of glutamate increases the level of MepM, but not MepS. These results indicate that the protein levels of MepS and MepM are tightly controlled by two distinct amino acid availability-dependent regulatory mechanisms.

Prc is a periplasmic protease that degrades MepS. Here, we unveiled that MepM is another substrate of Prc and that the proteolysis of MepM is not enhanced by NlpI. Both MepS and MepM are substrates of Prc, but the difference in the MepS expression between LB and minimal media was entirely mediated by Prc, whereas the difference in the MepM expression was hardly affected by Prc (Figure 4). This difference may be partly due to the role of NlpI. *In vitro* proteolysis experiments showed that the proteolysis rate of MepM by Prc alone was significantly lower than that of MepS by the combination of Prc and NlpI, indicating that the protein level of MepS is more strongly governed by Prc. Although the protein level of MepM was affected by Prc in the LB medium (Figure 3D), its level in the minimal medium seems to be strongly governed by other unknown regulatory mechanisms, but not by Prc. A recent report showed that MltG, one of lytic transglycosylases involved in the biogenesis of PG, is a substrate of Prc in *E. coli* (Hsu et al., 2020). Notably, like MepM, the proteolysis of MltG by Prc is also independent on NlpI. In *E. coli*, identified substrates of Prc are MepS, PBP3, MltG, and MepM, which are associated with PG biosynthesis. Therefore, Prc seems to play a regulatory function in the control of PG synthesis.

MepS and MepM show distinct mechanisms in the regulation of protein levels, despite being subjected to amino acid-mediated regulation. The levels of MepS and MepM were regulated by aromatic amino acids and glutamate, respectively (Figures 5, 6). The decreased level of MepS in the minimal medium was dependent on Prc, but that of MepM was not. Transcriptional patterns of the *mepS* and *mepM* genes were also significantly different (Figure 2A). Transcription of the *mepM* gene was strongly activated in the minimal medium despite the significant decrease in the protein level. However, transcription of the *mepS* gene was not activated in the minimal medium (Figure 2A). As various factors are different between LB and minimal media, the increase in *mepM* transcription in the minimal medium may be induced by other factor(s), not amino acids. Because this study is focused on the regulatory mechanisms of the MepS and MepM protein levels according to amino acid availability, we will investigate this issue in future studies.

In the LB medium, bacteria use amino acids as a nitrogen source, whereas they use ammonia in the minimal medium (Lu et al., 2013; Bren et al., 2016). The M9 minimal medium containing glucose is not subjected to nitrogen starvation. Glutamine concentration between the minimal medium and the amino acid-containing medium was almost similar (Schumacher et al., 2013), and in our study, the addition of glutamine did not affect the protein levels of either MepS or MepM (Figures 5A,B). Additionally, a deletion of RpoN, a sigma factor involved in nitrogen assimilation, did not affect the expression of MepM (Supplementary Figure 9A). These results indicate that nitrogen assimilation, i.e., glutamine availability, was not associated with the levels of MepS and MepM. The cellular concentration of an alarmone (p)ppGpp did not affect the expression of MepM. The expression pattern of MepM did not change with the deletion of RelA, which is responsible for the accumulation of (p)ppGpp under amino acid starvation (Supplementary Figure 9B). The level of (p)ppGpp was very low in the minimal medium containing sufficient ammonia concentrations (Lee et al., 2018), as in the LB medium. Collectively, all these results demonstrated that nitrogen or amino acid starvation did not induce a decrease in MepS and MepM.

In the M9 minimal medium containing glucose, cells do not encounter nitrogen or amino acid starvation, but they should synthesize all amino acids via de novo pathways, unlike in the LB medium. This difference could affect the cellular levels of PG precursors that are required for PG synthesis. Because PG is composed of amino sugars and amino acids, a sufficient supply of amino acids from the extracellular medium could increase the intracellular levels of PG precursors. For example, the cellular level of N-acetylglucosamine is regulated by a multifunctional small RNA binding protein, RapZ, which senses the cellular level of glucosamine-6-phosphate necessary for N-acetylglucosamine biosynthesis (Khan and Gorke, 2020; Khan et al., 2020). Because RapZ post-transcriptionally controls the enzyme GlmS, which is responsible for glucosmine-6-phosphate biosynthesis (Khan and Gorke, 2020), it could be a regulator of MepM levels. However, the deletion of RapZ did not affect the expression pattern of MepM (Supplementary Figure 9C). Although the presence of aromatic amino acids and glutamate strongly affects the levels of MepS and MepM, respectively, we do not know why aromatic amino acids and glutamate are related to the protein levels of PG endopeptidases. Because aromatic amino acids are high cost amino acids (Zampieri et al., 2019), the presence of aromatic amino acids could alleviate the burden of amino acid biosynthesis and could increase the biosynthesis of PG precursors, which may require increased levels of PG endopeptidases for enhancing the activity of PG biosynthesis. The reason why histidine affects the growth of the *mepS mepM* mutant is also unknown (Supplementary Figure 8B). Therefore, further experiments are required to investigate the in-depth mechanisms of specific amino acid availability-dependent regulation of MepS and MepM.

**Figure 7:**
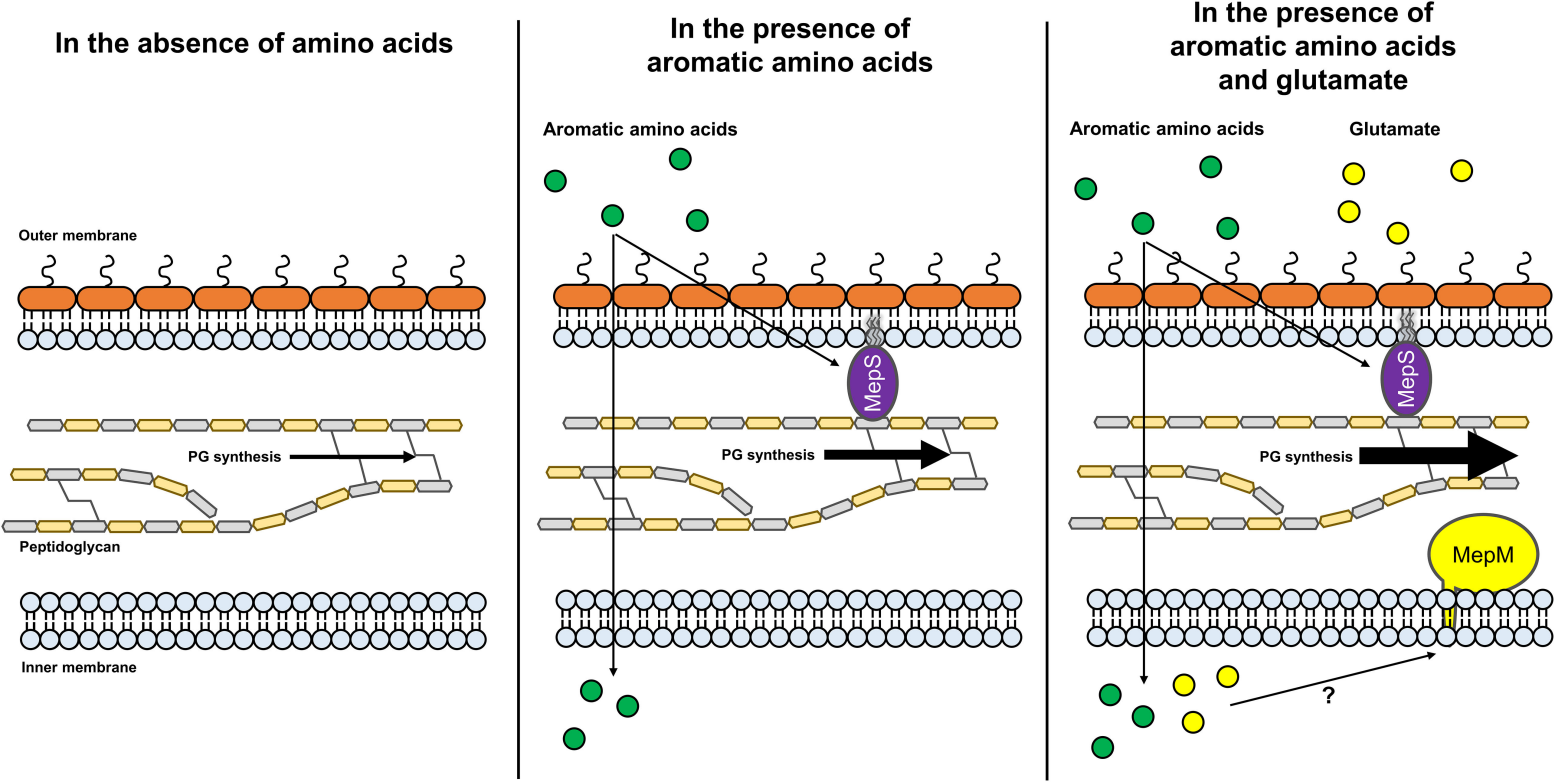
A model for amino acid-dependent regulation of the protein levels of MepS and MepM. Under low amino acid availability, the protein level of MepS decreases through a Prc-mediated mechanism. Through an unknown regulatory mechanism, the protein level of MepM also decreases. The decreased levels of MepS and MepM may diminish the synthesis of PG. In the presence of aromatic amino acids, the protein level of MepS increases, whereas the protein level of MepM remains low. The increased protein level of MepS may slightly enhance the synthesis of PG. In the presence of aromatic amino acids and glutamate, the protein levels of both MepS and MepM increase. Increased protein levels of MepS and MepM may strongly enhance the synthesis of PG. Green and yellow circles indicate aromatic amino acids and glutamate, respectively.

In this study, we unveiled that MepS and MepM, which are necessary for bacterial growth under amino acid-rich conditions, are regulated by amino acid availability. Moreover, we showed that the detailed regulatory mechanisms of MepS and MepM are distinct. We presented a model for the regulatory mechanisms of the expression of two PG endopeptidases (Figure 7). This study is the first to present a link between PG biosynthesis and nutrient conditions, which improves our understanding of the regulation of bacterial PG biosynthesis.

## Data Availability Statement

The original contributions presented in the study are included in the article/Supplementary Material, further inquiries can be directed to the corresponding author/s.

## Author Contributions

C-RL contributed to the conception and the design of experiments, and YK, BC, SP, HL, JS, UC, W-JC, and C-RL researched and wrote the manuscript. All authors contributed to the article and approved the submitted version.

## Conflict of Interest

The authors declare that the research was conducted in the absence of any commercial or financial relationships that could be construed as a potential conflict of interest.
