# Supplementary material for: Distinct Amino Acid Availability-Dependent Regulatory Mechanisms of MepS and MepM Levels in Escherichia coli
Source: Front Microbiol. 2021 Jun 30;12:677739. doi: 10.3389/fmicb.2021.677739 (PMC8278236; doi:10.3389/fmicb.2021.677739)
Supplement: Supplementary file 1 [file Table_1.docx]

Supplementary Material

# Supplementary Figures and Tables

## Supplementary Figures


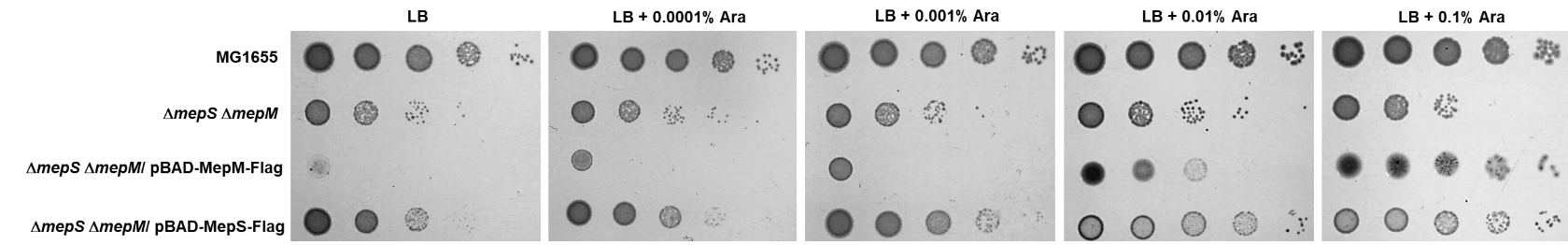


**Supplementary Figure 1.** Complementation of the phenotype of the *mepS mepM* double mutant. The cells of indicated strains were serially diluted from 10^8^ to 10^4^ cells/ml in 10-fold steps and spotted onto an LB plate or LB plates containing indicated concentrations of arabinose (Ara).


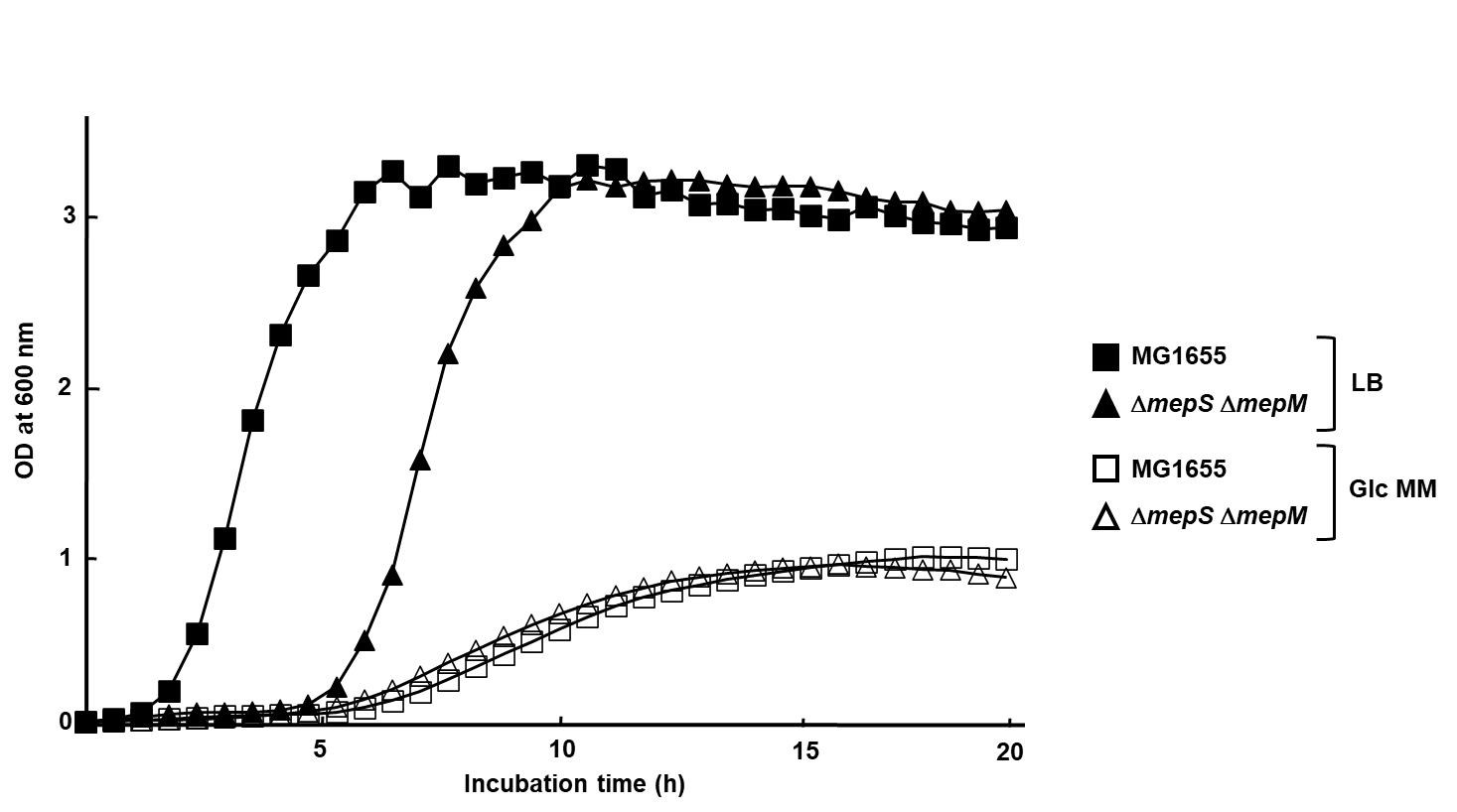


**Supplementary Figure 2.** Growth curves of the *mepS mepM* double mutant. The indicated cells grown in LB overnight were inoculated in LB medium or M9 minimal medium containing 0.2% glucose (Glc MM). Cell growth was recorded by measuring the optical density at 600 nm: closed squares, MG1655 in LB medium; closed triangles, Δ*mepS* Δ*mepM* in LB medium; open squares, MG1655 in M9 minimal medium containing 0.2% glucose; and open triangles, Δ*mepS* Δ*mepM* in M9 minimal medium containing 0.2% glucose.


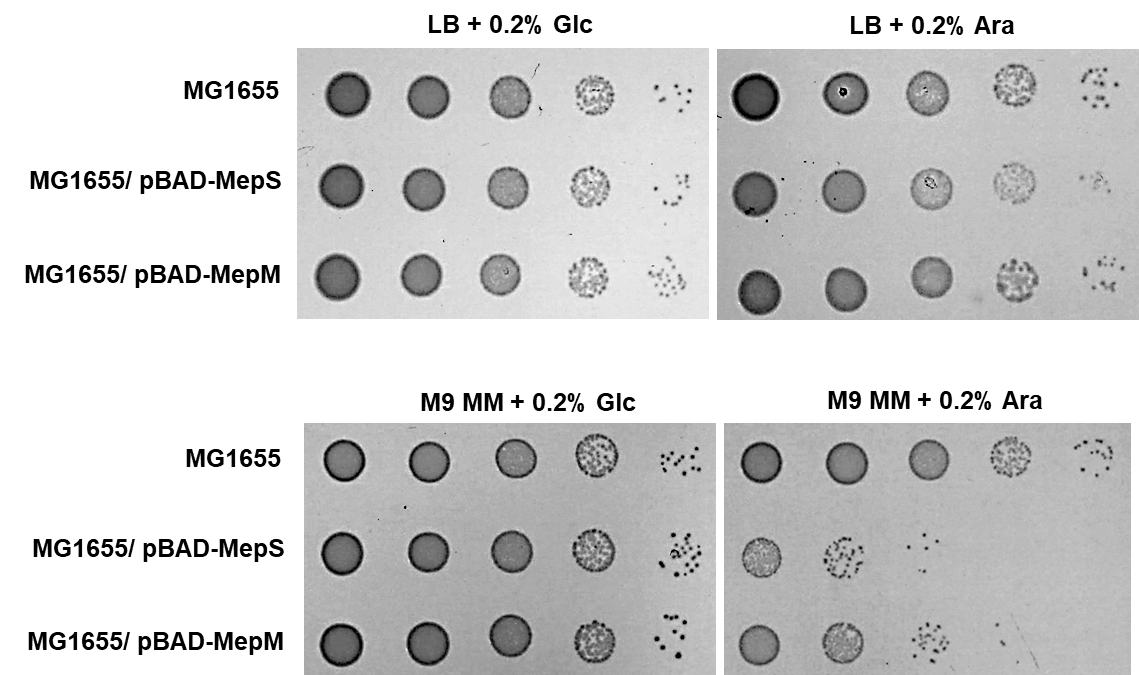


**Supplementary Figure 3.** Effect of overexpression of MepS and MepM. Indicated strains were serially diluted from 10^8^ cells/mL to 10^4^ cells/mL in 10-fold steps and spotted onto LB plates containing 0.2% glucose (Glc) or 0.2% arabinose (Ara) or M9 minimal medium (M9 MM) plates containing 0.2% glucose or 0.2% arabinose.


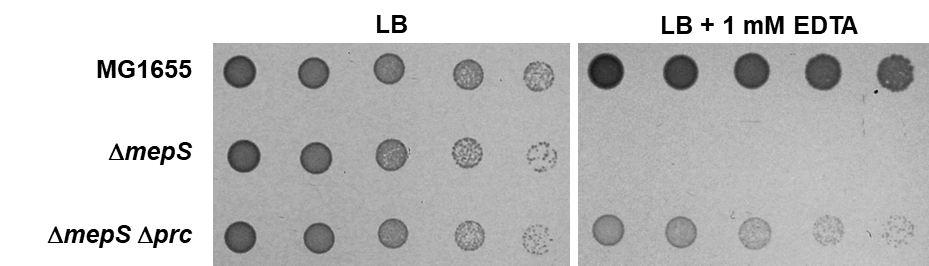


**Supplementary Figure 4.** Partial suppression of EDTA sensitivity of the *mepS* mutant by the deletion of Prc. Indicated strains were serially diluted from 10^8^ cells/mL to 10^4^ cells/mL in 10-fold steps and spotted onto an LB plate or an LB plate containing 1 mM EDTA.


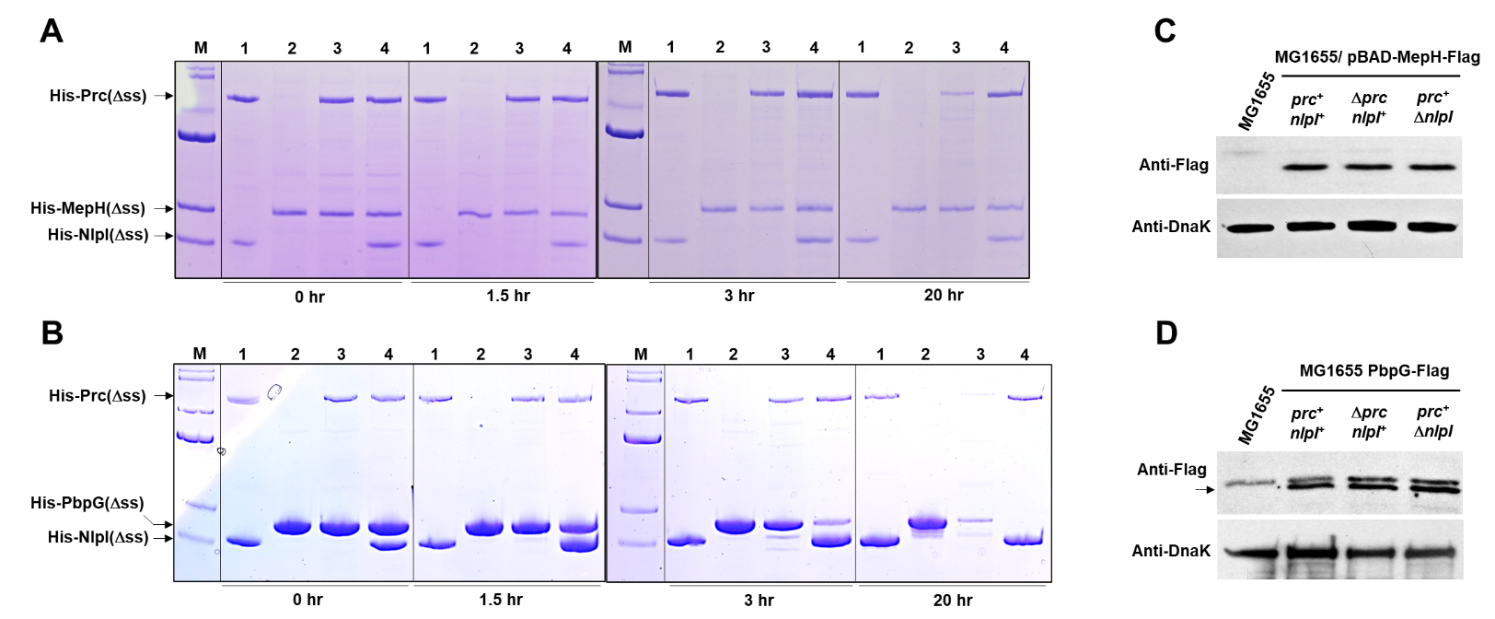


**Supplementary Figure 5.** MepH and PbpG are not a substrate of Prc. **(A,B)** *In vitro* proteolysis assay of MepH and PbpG by Prc. Purified MepH **(A)** or PbpG **(B)** was incubated with Prc in the presence or the absence of NlpI at 37°C for the indicated times. The samples were analyzed by SDS-PAGE and stained with Coomassie Brilliant Blue R. Lane M, EzWayTM Protein Blue MW Marker (KOMA Biotech., Korea); lane 1, His-Prc(Δss) and His-NlpI(Δss); lane 2, His-MepH(Δss) or His-PbpG(Δss); lane 3, His-Prc(Δss) and His-MepH(Δss) or His-PbpG(Δss); lane 4, His-Prc(Δss), His-NlpI(Δss), and His-MepH(Δss) or His-PbpG(Δss). **(C,D)** Intracellular levels of MepH and PbpG. The indicated strains were grown in LB medium to exponential phase (OD_600nm_=0.8). Harvested cells (5 x 10^7^ cells) were used to determine the intracellular levels of MepH **(C)** and PbpG **(D)** using an anti-Flag antibody. DnaK was used as the loading control.


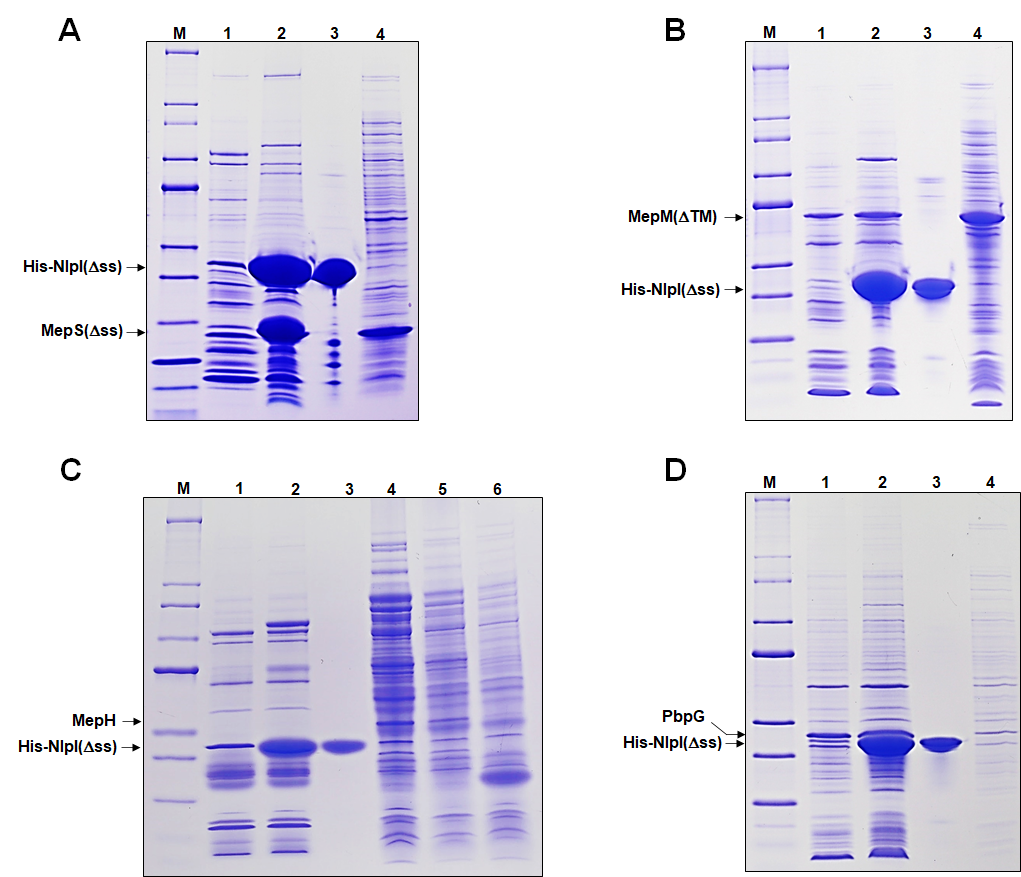


**Supplementary Figure 6.** Pull-down assay to assess the *in vitro* interaction between NlpI and PG endopeptidases. Purified His-NlpI(Δss) was used as a bait for investigating the interaction with PG endopeptidases, including MepS **(A)**, MepM **(B)**, MepH **(C)**, and PbpG **(D)**. Purified His-NlpI(Δss) was mixed with the soluble supernatant of PG endopeptidase-overexpressing cell lysates and His-NlpI(Δss) was pull-downed using a Talon metal affinity resin. Interaction partner of NlpI was detected in the sample containing His-NlpI(Δss). Lane M, EzWayTM Protein Blue MW Marker (KOMA Biotech., Korea); lane 1, pull-down sample from the soluble supernatants of PG endopeptidase-overexpressing cell lysates; lane 2, pull-down sample from the mixture of purified NlpI(Δss) and the soluble supernatants of PG endopeptidase-overexpressing cell lysates; lane 3, purified NlpI(Δss); lane 4, the soluble supernatants of PG endopeptidase-overexpressing cell lysates; lane 5, MepH-overexpressing cells after IPTG induction; lane 6, MepH-overexpressing cells before IPTG induction.


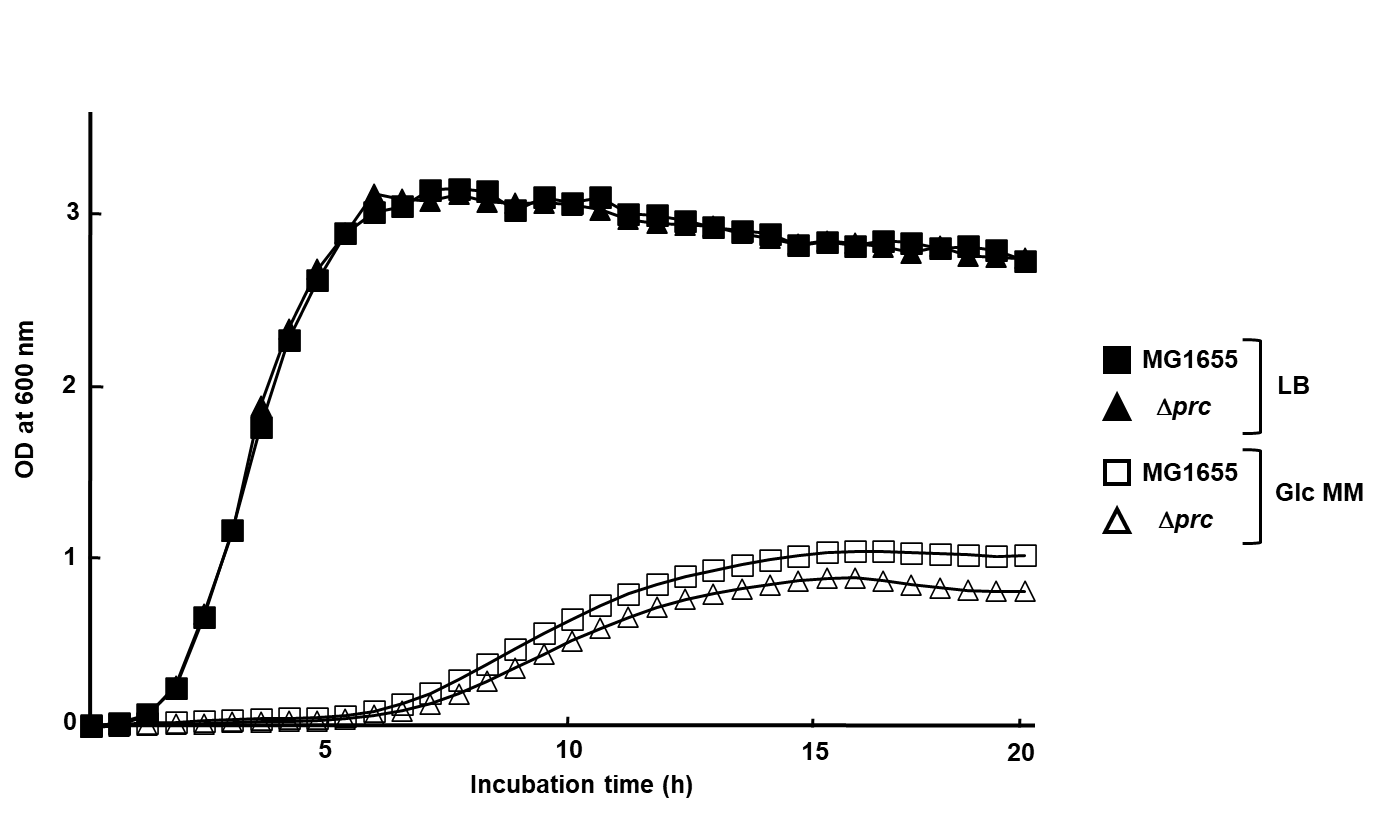


**Supplementary Figure 7.** Growth curves of the *prc* mutant. The indicated cells grown in LB overnight were inoculated in LB medium or M9 minimal medium containing 0.2% glucose (Glc MM). Cell growth was recorded by measuring the optical density at 600 nm: closed squares, MG1655 in LB medium; closed triangles, Δ*prc* in LB medium; open squares, MG1655 in M9 minimal medium containing 0.2% glucose; and open triangles, Δ*prc* in M9 minimal medium containing 0.2% glucose.


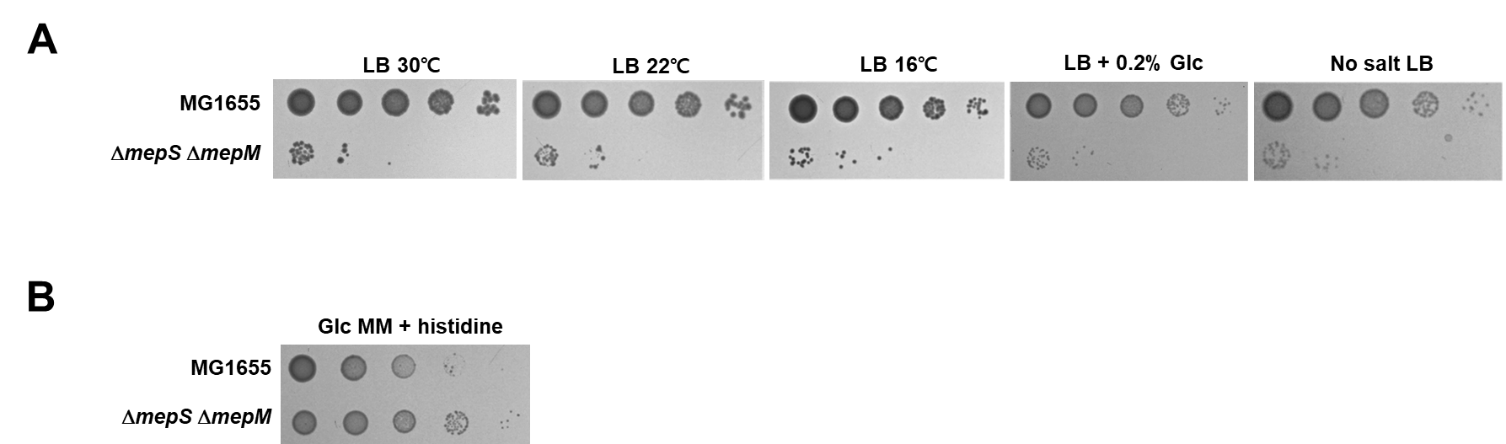


**Supplementary Figure 8.** Phenotypes of the *mepS mepM* double mutant. (**A**) Effects of temperature, glucose, and salt on the growth of the *mepS mepM* double mutant. Indicated strains were serially diluted from 10^8^ cells/mL to 10^4^ cells/mL in 10-fold steps and spotted onto LB plates, an LB plate containing 0.2% glucose (Glc), or an LB plate without NaCl (No salt LB). (**B**) Effect of the addition of histidine on the growth of the *mepS mepM* double mutant in M9 minimal medium. Indicated strains were serially diluted from 10^8^ cells/mL to 10^4^ cells/mL in 10-fold steps and spotted onto a glucose M9 minimal medium (Glc MM) plate containing histidine (10 mM).


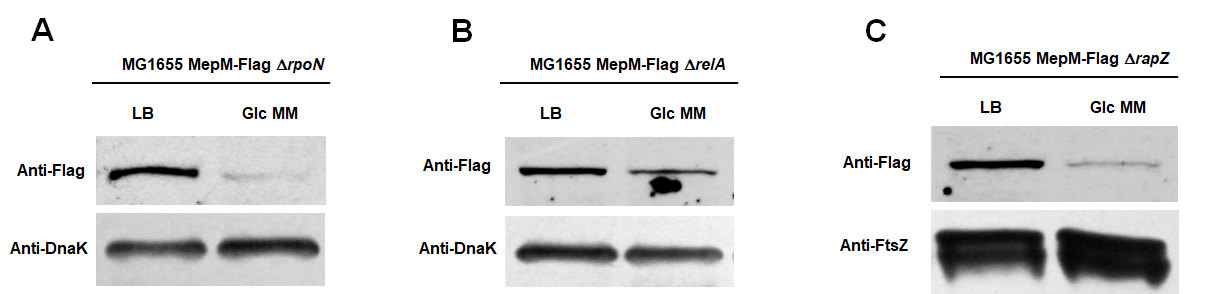


**Supplementary Figure 9.** Effect of deletions of the *rpoN*, relA, and rapZ genes on the protein level of MepM. The indicated strains were grown in LB or M9 minimal medium containing glucose (Glc MM) to early exponential phase. Harvested cells (5 x 10^8^ cells) were used to determine the intracellular levels of MepM using an anti-Flag antibody. DnaK and FtsZ were used as the loading controls.

## Supplementary Tables

**Supplementary Table 1.** ***Escherichia coli* strains and plasmids used in this study.**

| Strain or plasmid | Genotype or phenotype | Source or Reference |
| --- | --- | --- |
| **Strains** |  |  |
| MG1655 | F^-^ λ^-^ *ilvG*^-^ *rfb*-50 *rph*-1. Wild type *E. coli* K-12 | (Blattner et al., 1997) |
| ER2566 | *F-l-fhuA2 [lon] ompT lacZ::T7 p07 gene gal sulA11* Δ*(mcrC-mrr)114::IS10*  *R(mcr-73::miniTn10-TetS)2*  *R(zgb-210::Tn10)(tetS) endA1 [dcm]* | New England Biolabs |
| MG1655 Δ*mepM* | MG1655 *mepM::frt* | (Park et al., 2020) |
| MG1655 Δ*mepS* | MG1655 *mepS::frt* | (Park et al., 2020) |
| MG1655 Δ*prc* | MG1655 *prc::frt* | This study |
| MG1655 Δ*mepS* Δ*prc* | MG1655 *prc::frt mepS::Kan*^R^ | This study |
| MG1655 Δ*mepM* Δ*mepS* | MG1655 *mepM::frt mepS::Kan*^R^ | (Park et al., 2020) |
| MG1655 Δ*mepM* Δ*mepH* | MG1655 *mepM::frt mepH::*Kan^R^ | This study |
| MG1655 Δ*mepM* Δ*pbpG* | MG1655 *mepM::frt pbpG::*Kan^R^ | This study |
| MG1655 Δ*mepS* Δ*mepH* | MG1655 *mepS::frt mepH::*Kan^R^ | (Park et al., 2020) |
| MG1655 Δ*mepS* Δ*pbpG* | MG1655 *mepS::frt pbpG::*Kan^R^ | This study |
| MG1655 Δ*mepH* Δ*pbpG* | MG1655 *pbpG::frt mepH::*Kan^R^ | This study |
| MG1655 *mepM*-3xFLAG | MG1655 *mepM*-3xFLAG, Cm^R^ | (Park et al., 2020) |
| MG1655 *mepS*-3xFLAG | MG1655 *mepS*-3xFLAG, Cm^R^ | This study |
| MG1655 *pbpG*-3xFLAG | MG1655 *pbpG*-3xFLAG, Cm^R^ | This study |
| MG1655 *mepM-*3xFLAG Δ*prc* | MG1655 *mepM*-3xFLAG, Cm^R^ *prc::*Kan^R^ | This study |
| MG1655 *mepM-*3xFLAG Δ*nlpI* | MG1655 *mepM*-3xFLAG, Cm^R^ *nlpI::*Kan^R^ | This study |
| MG1655 *mepM-*3xFLAG Δ*relA* | MG1655 *mepM*-3xFLAG, Cm^R^ *relA::*Kan^R^ | This study |
| MG1655 *mepM-*3xFLAG Δ*rpoN* | MG1655 *mepM*-3xFLAG, Cm^R^ *rpoN::*Kan^R^ | This study |
| MG1655 *mepM-*3xFLAG Δ*rapZ* | MG1655 *mepM*-3xFLAG, Cm^R^ *rapZ::*Kan^R^ | This study |
| MG1655 *mepS-*3xFLAG Δ*prc* | MG1655 *mepS*-3xFLAG, Cm^R^ *prc::*Kan^R^ | This study |
| MG1655 *mepS-*3xFLAG Δ*nlpI* | MG1655 *mepS*-3xFLAG, Cm^R^ *nlpI::*Kan^R^ | This study |
| **Plasmids** |  |  |
| pBAD24 | Expression vector under control of arabinose-inducible promoter, Amp^R^ | Addgene |
| pBAD24(cm) | pBAD24-based expression vector, Cm^R^ | (Park et al., 2020) |
| pET24a | Expression vector under control of T7 promoter, Kan^R^ | Novagen |
| pET28a | Expression vector under control of T7 promoter, Kan^R^ | Novagen |
| pBAD-MepM-Flag | pBAD24(Cm)-based expression vector for fused with a 3X FLAG tag at the C-terminus, Cm^R^ | (Park et al., 2020) |
| pBAD-MepS-Flag | pBAD24(Cm)-based expression vector for MepS fused with a 3X FLAG tag at the C-terminus, Cm^R^ | (Park et al., 2020) |
| pBAD-MepH-Flag | pBAD24(Cm)-based expression vector for MepH fused with a 3X FLAG tag at the C-terminus, Cm^R^ | (Park et al., 2020) |
| pBAD-MepH | pBAD24(cm)-based expression vector for MepH, Cm^R^ | (Park et al., 2020) |
| pET28a-MepM(ΔTM) | pET28a-based expression vector for MepM(ΔTM, Δ1-40) with N-terminal 6 histidines, Kan^R^ | (Park et al., 2020) |
| pET28a-MepS(Δss) | pET28a-based expression vector for MepS(Δss, Δ1-27) with N-terminal 6 histidines, Kan^R^ | (Park et al., 2020) |
| pET28a-MepH(Δss) | pET28a-based expression vector for MepH(Δss, Δ1-27) with N-terminal 6 histidines, Kan^R^ | This study |
| pET28a-PbpG(Δss) | pET28a-based expression vector for PbpG(Δss, Δ1-29) with N-terminal 6 histidines, Kan^R^ | This study |
| pET28a-Prc(Δss) | pET28a-based expression vector for Prc(Δss, Δ1-22) with N-terminal 6 histidines, Kan^R^ | This study |
| pET28a-NlpI(Δss) | pET28a-based expression vector for NlpI(Δss, Δ1-20) with N-terminal 6 histidines, Kan^R^ | This study |
| pET24a-MepM(ΔTM) | pET24a-based expression vector for MepM(ΔTM, Δ1-40), Kan^R^ | This study |
| pET24a-MepS(Δss) | pET24a-based expression vector for MepS(Δss, Δ1-27), Kan^R^ | This study |
| pET24a-PbpG | pET24a-based expression vector for PbpG, Kan^R^ | This study |

**Supplementary Table 2.** **Oligonucleotides used in this study**

| **Name** | **Oligonucleotide sequence (5’–3’)** | **Use(s)** |
| --- | --- | --- |
| d*mepM*-FRT-F | CCAACCAGTATGCGAGCTGCCTGAAAGGAGATTAATGAGGAAGTGATTACGTGTAGGCTGGAGCTGCTTC | Deletion |
| d*mepM*-FRT-R | GACGAGATACGGAACTGTTTCGCCGTCGGGAATCGCAAGAATCCTTTCGCATTCCGGGGATCCGTCGACC |  |
| d*mepS*-FRT-F | TTTTTTATAACGATATTTGTCGTTAAGGACTTCAAGGGAAAACAAACAACGTGTAGGCTGGAGCTGCTTC |  |
| d*mepS*-FRT-R | CTTTCAGTTAACTCGTCAGGATAGCCAAGGGATTGCATCCAAACGGTTTAATTCCGGGGATCCGTCGACC |  |
| d*mepH*-FRT-F | TGTAAATCAGACGCAGGCATGATAGACCTGCCTTTACAGAGGGACGCTCAGGTAGGCTGGAGCTGCTTC |  |
| d*mepH*-FRT-R | GCCGACATACACGCCGACATGATCGGCTGTGCCGCGTCCCTGAGTACGGAATTCCGGGGATCCGTCGACC |  |
| d*pbpG*-FRT-F | CTCCGGCGGTGCGCAACCCGTGCGCGTGAACCACTATCTGAATGCTCATCGTGTAGGCTGGAGCTGCTTC |  |
| d*pbpG*-FRT-R | ATTTGCCAAACGCGTCCATCACTACCAGCGCCACCGGTTTATTATTGATAATTCCGGGGATCCGTCGACC |  |
| d*prc*-FRT-F | AAACGGAGGCCGGGCCAGGCATGAACATGTTTTTTAGGCTTACCGCGTTAGTGTAGGCTGGAGCTGCTTC |  |
| d*prc*-FRT-R | ATCTTGTCGCTGTTAAAAAATCAGGCACAATTTCTTGTGCCTGATTGATAATTCCGGGGATCCGTCGACC |  |
| d*nlpI*-FRT-F | AGCAACCGGGAACAGGACGTTCATTCAACCGTGGTCTTCGGGAGTGGGAAGTGTAGGCTGGAGCTGCTTC |  |
| d*nlpI*-FRT-R | AGACTTAGGTAGTACTTACCTAAATGAAGTTGGTTTCACTGAGATGCTCATTCCGGGGATCCGTCGACC |  |
| d*relA*-FRT-F | GGAGAGGACGATGGTTGCGGTAAGAAGTGCACATATCAATAAGGCTGGTGGTGTAGGCTGGAGCTGCTTC |  |
| d*relA*-FRT-R | TACACCCGGTCGTCAAAGACCTGACTACGTACTTCGTCGAGCATTTCGCCATTCCGGGGATCCGTCGACC |  |
| d*rpoN*-FRT-F | GATTCTGAACATGAAGCAAGGTTTGCAACTCAGGCTTAGCCAACAACTGGGTGTAGGCTGGAGCTGCTTC |  |
| d*rpoN*-FRT-R | TCGAGCGACTGGATCAGATTGACGGCTTCTTTCAGCACATCTTCTTTCAGATTCCGGGGATCCGTCGACC |  |
| d*rapZ*-FRT-F | TCTTTTAGACGTTGTGAGGAGAAACAGTACATGGTACTGATGATCGTCAGGTGTAGGCTGGAGCTGCTTC |  |
| d*rapZ*-FRT-R | TAACCATAGCTCAAGATAGCTTCGCGTCTGGTAGATAAAATTGTGTACTTATTCCGGGGATCCGTCGACC |  |
| d*mepM*-cfm-F | CAAGCTATTCAGAATTCCTGAGTCAATTAG | Deletion  cfm |
| d*mepM*-cfm-R | ACCGGATTAGTACGACGCGGGTTAAAATTA |  |
| d*mepS*-cfm-F | CCAGGTAATTAGTCTCGTGTCGCTTGGCAT |  |
| d*mepS*-cfm-R | TGAAAACAAAAAAGCACTGCCTAAGCAGTG |  |
| d*mepH*-cfm-F | CTTCTTCTATGCATTAGAATCATCAAGTTT |  |
| d*mepH*-cfm-R | TGTGCGCGGTGACTGAATAAATTTGCCGTT |  |
| d*pbpG*-cfm-F | GATGGCGTCGCTAGCCTCAGTAAATCCTTA |  |
| d*pbpG*-cfm-R | GCAGGCGGCTGGCATCGGCAAAATGGGTAT |  |
| d*prc*-cfm-F | GTATGTCTTTGATTGTGCGCGCAGAACACC |  |
| d*prc*-cfm-R | TTGTAGCATCTGATTTACGGCATCTTGTCG |  |
| d*nlpI*-cfm-F | CAGGGCGAGTAAGGTTGCCATTTGCCCTCC |  |
| d*nlpI*-cfm-R | CGGGCTGATGTGTACGTCAGCTATTGCTGG |  |
| d*relA*-cfm-F | TCCCACACACGGGACATCTGGAATCGATGG |  |
| d*relA*-cfm-R | GTGGTAAGCGAAGTCCAGCGGCGTTGATCC |  |
| d*rpoN*-cfm-F | CTTCAGACTCTGATAGGGTAGAAGTTTGCG |  |
| d*rpoN*-cfm-R | AGCACATCTGGAATGACATACTCAGGTTCG |  |
| d*rapZ*-cfm-F | GCGCAGCCCAGAGCGATGAAGAGCTGTATC |  |
| d*rapZ*-cfm-R | CAACCAATGGCGACCGTCAAGTAGCTACGG |  |
| Chromosomal *mepM*-FLAG-insert-F | AGCAGCGTTGAGCTATAAAAAACAAAAAGCCGCCCAAATGGCGGCGGCGGGGCAGACGGCACAGAACGATGTCGACCTGCAGGATTATAAG | Chromosomal insertion |
| Chromosomal *mepM*-FLAG-insert-R | CCAGTAAACGGAAAAACTGGCAGGAAGTGGAGTAAAAATTACGGATGGCAGAGTATCGCCATCCGAATTCTTACGCCCCGCCCTGCCACT |  |
| Chromosomal *mepS*-FLAG-insert-F | TATTTCCAGCATGAATGAACCGTACTGGAAGAAGCGTTACAACGAAGCACGCCGGGTTCTCAGCCGCAGCGTCGACCTGCAGGATTATAAG |  |
| Chromosomal *mepS*-FLAG-insert-R | AAAGCACTGCCTAAGCAGTGCTTTCAGTTAACTCGTCAGGATAGCCAAGGGATTGCATCCAAACGGTTTATTACGCCCCGCCCTGCCACT |  |
| Chromosomal *mepH*-FLAG-insert-F | AACTGAAAAACGGCGACCTGGTCTTTTTCCGTACTCAGGG |  |
| Chromosomal *mepH*-FLAG-insert-R | ATAGGGTAGCAAATTAAAAGGTGAGACAAAAGATGAACTTACCCTGTTGCCGTAACAACAGGGTAAAGTTTTACGCCCCGCCCTGCCACT |  |
| Chromosomal *pbpG*-FLAG-insert-F | AGCAGCGTTGAGCTATAAAAAACAAAAAGCCGCCCAAATGGCGGCGGCGGGGCAGACGGCACAGAACGATGTCGACCTGCAGGATTATAAG |  |
| Chromosomal *pbpG*-FLAG-insert-R | CCAGTAAACGGAAAAACTGGCAGGAAGTGGAGTAAAAATTACGGATGGCAGAGTATCGCCATCCGAATTCTTACGCCCCGCCCTGCCACT |  |
| Chromosomal *mepM*-FLAG-cfm-F | CGTTATATGCACTTGCGCAAGATTCTGGTG | Chromosomal insertion  cfm |
| Chromosomal *mepM*-FLAG-cfm-R | GTTTCCATGCTTTTCCAGTTTCGGATAAGG |  |
| Chromosomal *mepS*-FLAG-cfm-F | AGGATGAATTTGAAAACCTGGTTCGTAATG |  |
| Chromosomal *mepS*-FLAG-cfm-R | TGACCGGACTATAGCCTGGATCAAGACGCG |  |
| Chromosomal *mepH*-FLAG-cfm-F | GTATTCCGCGTACGGCGAATGAAATGTATC |  |
| Chromosomal *mepH*-FLAG-cfm-R | ATACGTACAATAGCCTTATTGTGCGTATGG |  |
| Chromosomal *pbpG*-FLAG-cfm-F | CCGGCTTTACCAATGCGGCGGGCCATGTC |  |
| Chromosomal *pbpG*-FLAG-cfm-R | TCCGACCAACAGCATGAATCCGGGGATCGG |  |
| pBAD-MepH-Flag insert-F | CTAGCAGGAGGAATTCATGGCGCGGATAAACCG | Cloning |
| pBAD-MepH-Flag insert-R | AATCCTGCAGGTCGACGCGAAGTGTTTTTGGGGTCA |  |
| pBAD-MepM-Flag insert-F | CTAGCAGGAGGAATTCATGCAACAGATAGCCCGCTC |  |
| pBAD-MepM-Flag insert-R | AATCCTGCAGGTCGACATCAAACCGTAGCTGCGGCA |  |
| pET28a-MepH insert-F | CGCGCGGCAGCCATATGTCAAAGCAAGCCAGGGAGAG |  |
| pET28a-MepH insert-R | GCTCGAATTCGGATCCCTGTTGCCGTAACAACAGGG |  |
| pET28a-PbpG insert-F | CGCGCGGCAGCCATATGGCTACCACCGCTTCACAACC |  |
| pET28a-PbpG insert-R | GCTCGAATTCGGATCCTGGCAGAGTATCGCCATCCG |  |
| pET28a-Prc insert-F | CGCGCGGCAGCCATATGAACATGTTTTTTAGGCTTAC |  |
| pET28a-Prc insert-R | GCTCGAATTCGGATCCCCTGATTGATATTACTTGAC |  |
| pET28a-NlpI insert-F | CGCGCGGCAGCCATATGAAGCCTTTTTTGCGCTG |  |
| pET28a-NlpI insert-R | GCTCGAATTCGGATCCAAAGATTACGGGCTGATGTG |  |
| pET28a-MepM insert-F | CGCGCGGCAGCCATATGTATCACCGTGATGCCACGCC |  |
| pET28a-MepM insert-R | GCTCGAATTCGGATCCTGCGTACCGGCTGCGAATGG |  |
| pET24a-MepS insert-F | AAGGAGATATACATATGGTCAAATCTCAACCGAT |  |
| pET24a-MepS insert-R | GCTCGAATTCGGATCCTTAACTCGTCATTATAGCCA |  |
| pET24a-PbpG insert-F | AAGGAGATATACATATGCCGAAATTTCGAGTTTCTTT |  |
| pET24a-PbpG insert-R | GCTCGAATTCGGATCCTGGCAGAGTATCGCCATCCG |  |
| pBAD-cfm-F | TCGCAACTCTCTACTGTTTCTCCATACCCG |  |
| pBAD-cfm-R | CAAAACAGCCAAGCTGGAGACCGTTTAAAC |  |

| pET-cfm-F | TAATACGACTCACTATAGGG |  |
| --- | --- | --- |
| pET-cfm-R | GCTAGTTATTGCTCAGCGG |  |

| RT-16SrRNA-F | AAATTGAAGAGTTTGATCATGGCTCAGATT | qRT-PCR |
| --- | --- | --- |
| RT-16SrRNA-R | AATGAGCAAAGGTATTAACTTTACTCCCTT |  |
| RT-MepM-F | TAGCCCGCTCTGTCGCCCTGGCGTTTAATA |  |
| RT-MepM-R | TCAGGTTACGCAATTCTTTGTCGGCCGCAG |  |
| RT-MepS-F | TTTTGAGATATATCTTGCGCGGGATTCCCG |  |
| RT-MepS-R | AATCACCCGTACGCAAATTACTGCGGGAAA |  |

**References**

Blattner, F. R., Plunkett, G., 3rd, Bloch, C. A.*, et al.* (1997). The complete genome sequence of *Escherichia coli* K-12. *Science*. 277, 1453-1462. doi:

Park, S. H., Kim, Y. J., Lee, H. B., Seok, Y. J., Lee, C. R. (2020). Genetic evidence for distinct functions of peptidoglycan endopeptidases in *Escherichia coli*. *Front Microbiol*. 11, 565767. doi: 10.3389/fmicb.2020.565767
